# An Overview on the Catalytic Materials Proposed for the Simultaneous Removal of NO_x_ and Soot

**DOI:** 10.3390/ma13163551

**Published:** 2020-08-12

**Authors:** Lidia Castoldi

**Affiliations:** Dipartimento di Energia, Laboratory of Catalysis and Catalytic Processes and NEMAS, Centre of Excellence, Politecnico di Milano, via La Masa 34, 20156 Milano, Italy; lidia.castoldi@polimi.it

**Keywords:** soot, NO_x_, simultaneous removal, emission control, oxidation catalysts

## Abstract

Vehicular pollution has become a major problem in urban areas due to the exponential increase in the number of automobiles. Typical exhaust emissions, which include nitrogen oxides (NO_x_), hydrocarbons (HC), carbon monoxide (CO), soot, and particulate matter (PM), doubtless have important negative effects on the environment and human health, including cardiovascular effects such as cardiac arrhythmias and heart attacks, and respiratory effects such as asthma attacks and bronchitis. The mitigation measures comprise either the use of clean alternative fuels or the use of innovative technologies. Several existing emission control technologies have proven effective at controlling emissions individually, such as selective catalytic reduction (SCR) and lean NO_x_ trap (LNT) to reduce NO_x_ and diesel particulate filter (DPF) specifically for PM abatement. These after-treatment devices are the most profitable means to reduce exhaust emissions to acceptable limits (EURO VI norms) with very little or no impact on the engine performances. Additionally, the relative lack of physical space in which to install emissions-control equipment is a key challenge for cars, especially those of small size. For this reason, to reduce both volume and cost of the after-treatment devices integrated catalytic systems (e.g., a sort of a “single brick”) have been proposed, reducing both NO_x_ and PM simultaneously. This review will summarize the currently reported materials for the simultaneous removal of NO_x_ and soot, with particular attention to their nature, properties, and performances.

## 1. Introduction

In the theoretical or clean diesel combustion only CO_2_ and H_2_O are produced as exhaust, being carbon oxidized to CO_2_ and hydrogen to H_2_O. However, the oxidation process in actual diesel engines is very far from being an ideal process, so in the exhausts many byproducts and pollutants, both gaseous and solid, are present. Depending on many factors (air–fuel ratio, air–fuel concentration, ignition timing, turbulence in the combustion chamber, combustion form, combustion temperature, etc.) different exhaust compositions are obtained and a number of harmful products are generated; the most significant harmful products are CO, unburnt hydrocarbons (HC), NO_x_, and particulate matter (PM). To the vehicular pollution point of view, great importance related to NO_x_, CO, HC, and smoke (particles matters, PM, or soot), while CO_2_ emissions are mainly related to its greenhouse potential in the atmosphere.

To control and regulate the vehicular emissions, two regulatory commissions have been established, i.e., the Environmental Protection Agency (EPA) and the European Parliament (EURO) imposing the most stringent emission regulations; indeed, in Europe Euro VI protocols in force since 2015 have reduced emissions levels of NO_x_ by 87% and of PM by 96%. Many efforts have been made to develop adequate technologies to respect the imposed limits. Those are classified as primary or secondary techniques.

Diesel engines use highly compressed hot air to ignite the fuel. Air, mainly composed of oxygen and nitrogen, is initially drawn into the combustion chamber. Then, it is compressed, and the fuel is injected directly into this compressed air at about the top of the compression stroke in the combustion chamber. This generates high temperatures, which are sufficient for the diesel fuel to ignite spontaneously when it is injected into the cylinder. The high temperatures in the cylinders cause the nitrogen to react with oxygen and generate NO_x_ emissions, whose amount is a function of the maximum temperature in the cylinder, oxygen concentrations, and residence time. Most of the emitted NO_x_ is formed early in the combustion process, when the piston is still near the top of its stroke. This is when the flame temperature is the highest. So, it is an established factor that NO_x_ formation is highly dependent on temperature and reducing peak temperatures during combustion obviously reduce NO_x_.

To reduce the NO_x_ emissions from vehicles, there are two main approaches, i.e., first minimizing the amount of NO_x_ created, and second removing NO_x_ from the exhaust. The first task is mainly accomplished by manipulating engine operating characteristics (the so-called primary technology), for example by lowering the intake temperature, reducing power output, retarding the injector timing, reducing the coolant temperature, and/or reducing the combustion temperature. However, the problem of controlling NO_x_ in diesel exhaust is really complicated, and for this reason diesel vehicles require different approaches. Electronically controlled fuel injection, engine modification, increasing injection timing, water spray in the combustion chamber, improvement of fuel properties, use of fuel additives, etc., are some of the primary methods to reduce NO_x_ emissions. Between the other, cooled exhaust gas recirculation (EGR) is well known and is the method that most engine manufacturers are currently using. EGR system recycles a portion of the exhaust gas into the combustion chamber, mixing with fresh air and thus reducing the oxygen content and increasing the water vapor content of the combustion mixture; the result is a reduction of the peak combustion temperature and as consequence of NO_x_ formation. Unfortunately, the decreases in combustion temperature influences also the reaction rates, causing the emission of other pollutant species like CO, CO_2_, HC, and soot.

Reducing NO_x_ by manipulating engine operation generally reduces fuel efficiency. Besides, the mere manipulation of engine operation is not sufficient to meet the current stringent emission limits. As a result, after-treatment systems also need to be implemented, that remove the produced pollutant (mainly, NO_x_ and/or soot) from the exhausts.

The reduction of NO_x_ emissions is currently performed by selective catalytic reduction (SCR) [1] and lean NO_x_ traps (LNTs) [2] catalytic after-treatments. In the SCR system an aqueous urea solution (AdBlue^®^) is injected into the exhaust post combustion; the hydrolysis of urea generates ammonia, which reacts with the NO_x_ giving nitrogen and water. This catalytic reaction is accomplished by using zeolite-based catalysts, doped with Fe and/or Cu. With regard to the LNT system, NO_x_ are stored onto the catalyst surface during a lean phase (i.e., in the excess of oxygen), forming nitrites/nitrates ad-species; these are reduced to nitrogen during a subsequent rich phase (i.e., in the presence of a reductant like H_2_, CO, and HC) of a few seconds. In this case, the catalyst is a PGM-based system, i.e., a catalyst containing platinum-group metals (PGM) like Pt, Pd, and Rh, doped with alkaline and/or alkaline-earth metal oxides as storage components. The detailed mechanism of these systems will be addressed in Section 3.1.

**Figure 1:**
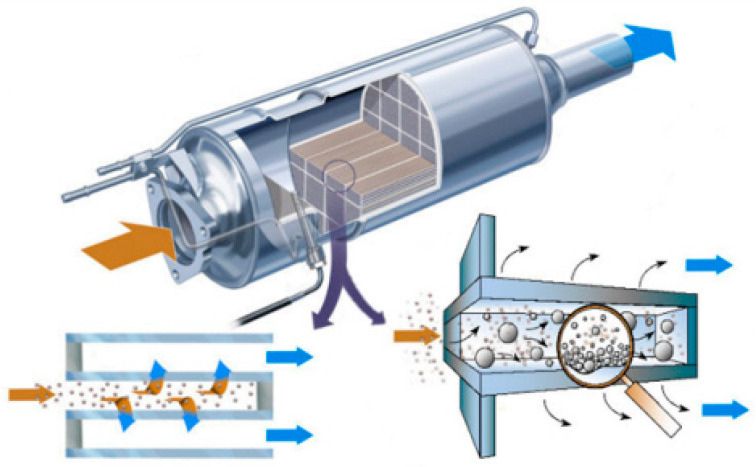
Functioning of a typical diesel particulate filter (DPF).

On the other hand, to meet EURO V regulations concerning soot all new vehicles must be equipped with a diesel particulate filter (DPF) that capture soot and other dangerous particles, preventing them releasing in the atmosphere. DPF are usually wall-flow monoliths in cordierite (2MgO–2Al_2_O_3_–5SiO_2_) or silicon carbide (SiC) with a honeycomb structure, where the channels are alternatively blocked at the end (Figure 1). A DPF can remove around 85% of the particulates from the exhaust.

If the filters are overloaded, the particles can cause obstruction of the flow of gas, which is manifested by an increase of the particulate filter pressure drop and as a consequence a decrease in the engine efficiency. The filter can operate until it is clogging; thus, it may be regenerated before the problem occurs.

During regeneration, the soot is oxidized into gaseous products. Thermal regeneration of DPF, also called active regeneration, requires a temperature of 550–600 °C, so that the controlled oxidation of the particulate with O_2_ occurs [3]. Active regeneration systems inject raw diesel fuel into the exhaust stream (post combustion) or into the diesel oxidation catalyst (DOC) to achieve appropriate regeneration temperatures in the DPF. Obviously, this process requires an extra fuel consumption leading to a fuel penalty; moreover, excessive heating can damage filter itself and the other downstream catalytic after-treatment devices.

The development of catalyzed DPFs (CDPFs), i.e., DPFs coated with a catalytic layer, aiming at lowering the soot oxidation temperature (passive regeneration) represents a viable solution to allow energy savings and prevent filter overheating during the regeneration phase. Accordingly, low temperature activity, on one hand, and high thermal stability, on the other hand, represent the main key issues for the development of suitable catalytic materials. Another option is to use the fuel-borne catalysts (FBC), i.e., active fuel additives constituted by metallic compounds (such as transition metals like cerium, iron, or copper compounds) that decrease the soot combustion temperature by improving the catalyst–soot contact [4,5]. However, several drawbacks such as the fuel penalty, possible FBC volatilization, formation of deposits on the DPF, and the high investment costs prevented the broad commercialization of FBCs.

An alternative commercially available technology is the continuously regenerating trap (CRT^®^) system, proposed by Johnson Matthey [6], which uses an oxidation diesel catalyst (DOC) upstream the DPF to generate NO_2_ (i.e., a stronger oxidant than O_2_ [7]) and consequently to decrease the soot combustion temperature in the downstream filter. Indeed, NO is oxidized to NO_2_ in the DOC, then the so-formed NO_2_ reacts with trapped soot, forming CO_2_ and NO, which in turn is reoxidized to NO_2_. The low NO_x_ removal efficiency, due to NO_2_ slip, represents the main problem of this technology [8].

Johnson Matthey further refined the CRT system by essentially combining CRT and CDPF in the so-called CCRT system (catalyzed continuously regenerating trap) where filter itself is coated with a catalyst, which improves the operating window for the filter regeneration.

Recently, to ensure the respect of the latest vehicular emission regulations, to reduce both packaging volume and cost of after-treatment systems, combined technologies have been also proposed to significantly remove PM and NO_x_ simultaneously. Accordingly, the use of combined DPF-LNT/SCR systems has been proposed in different configurations to take advantage of potential synergisms among the various devices, for example, to advantageously use concentration and/or temperature gradients generated by the catalytic system. Indeed, DPFs are fitted close to the engine where the exhaust is hottest so that passive regeneration is more likely to work; however, combining with LNT and/or SCR, the latter will be closer to the engine thus enjoying the advantage of higher temperatures for the NO_x_ removal.

Many types of catalysts have been investigated for the simultaneous control of NO_x_ and soot, i.e., PGM-based systems (platinum-group metals like Pt, Pd, Rh, and Ir), and also a wide variety of materilas like spinel-type oxides, hydrotalcites, rare earth metal oxides, mixed transient metal oxides, and perovskites. The high raw material cost of PGM catalysts has become a significant issue, so that the development of non-PGM catalysts is one of the most promising challenges.

In this context, the overall aim of this work is to give an overview on catalytic materials for the combined NO_x_ and soot removal. However, it is important to note that the concept of simultaneous removal of soot and NO_x_ is almost confused in the literature. First of all, the NO_x_ reduction expression is used in a double sense: NO_x_ reduction as NO_x_ concentration decrease, and reduction as formation of N-products with a lower oxidation state with respect to NO. Some authors refer to catalysts able to control simultaneously soot and NO_x_ because they are able to oxidize NO to NO_2_, which in turn is active in soot oxidation. In other cases, the simultaneous control is related to the catalysts ability in soot oxidation and NO_x_ storage without any deep study on the reduction mechanism of stored NO_x_. Finally, the simultaneous removal of NO_x_ and soot strictly speaking refers to oxidation of soot while NO_x_ are reduced to nitrogen. With this in mind, first of all we consider PGM-free catalysts that have been proposed for the simultaneous removal of soot and NO_x_, i.e., hydrotalcites differently doped and perovskite-like catalysts. Then, PGM-based catalysts have been considered; particular attention was paid to the DPNR (Diesel Particulate NOx Reduction) system patented by Toyota for the simultaneous removal of NO_x_ and soot. In addition to the traditional DPNR system based on traditional Pt-based NO_x_ storage-reduction catalyst, new PGM-free formulations have been deeply analyzed. Finally, with the aim of analyzing the combined technologies proposed to reduce simultaneously these pollutants and realize compact systems, SCR/or LNT/DPF systems have been considered and analyzed.

## 2. PGM-Free Catalysts for the Simultaneous Removal of Soot and NO_x_

### 2.1. Hydrotalcites and Mixed Metal Oxides Catalysts

Hydrotalcites (HTLCs) are part of the big family of anionic clays; they have Mg^2+^, Al^3+^, and CO_3_^2−^ ions as its constituents. These layered double hydroxides (LDHs) may be represented by the general formula [M_1−x_^II^ M_x_^III^(OH)_2_]_x_ + (A^n−^)_x/n_·yH_2_O, where M^2+^ (M^2+^ = Mg), M^3+^ (M^3+^ = Al) are di- and trivalent metal cations, A^n−^ is an interlayer anion (e.g., CO_3_^2−^) and x represents the molar fraction of M^3+^ per total metal. These materials can exhibit different physical and chemical properties depending on the nature of M^2+^ and/or M^3+^, on their molar ratios and on the interlayer anions. For these reasons, they are usually applied in catalysis [9], mainly for catalytic oxidation applications [10]. Since fresh hydrotalcites have high water content, which often makes them inactive for certain reactions, they are activated by thermal decomposition. The calcination temperature ranges from 500 to 800 °C, and the resultant mixed metal oxides after calcinations offer improved catalytic performances [11,12], exhibiting large surface area, basic properties, high metal dispersions, and low propensity to sintering; their redox properties could be further improved by doping with transition metal ions, i.e., Co, Cu, Ni, or Zn as M^2+^ cations, or Cr, Ga, Mn, or Fe as M^3+^ cations [11,13]; finally, the presence of alkali metals in the catalyst formulation enhances the catalytic soot combustion of hydrotalcite-derived materials [14,15,16,17,18].

LDHs represent suitable candidates for the simultaneous removal of NO_x_ and soot, as summarized in Table 1. Being their catalytic activity strongly related to the nature of metals, their amount and calcination temperature, different formulations have been proposed and between them copper-, cobalt-, or nickel-based oxides have demonstrated high activity in oxidation reactions [19], like soot combustion. Moreover, it is well known that the addition of alkali oxides, and in particular K can further improve their activities favoring the reaction through the formation of low melting point compounds [20], or of eutectics with other catalyst components, thus improving the surface mobility of the active species [12,13,21] and hence favoring the soot–catalyst contact, which has been claimed as a key factor in the soot oxidation process.

MgAl LDH is one of the most common precursors for synthesizing binary LDH-derived catalysts [9]. Zhang et al. [22,23,24] studied the performances in soot combustion of MgAl LDH derived mixed oxides eventually doped with K. Using a NO_x_/O_2_ mixture, the authors demonstrate that the ignition temperature (T_i_) and the temperature for 50% soot conversion (T_50_) decreased with the increase in the K loading, reaching the optimum of K below 8 wt % of the supporting amount. Moreover, also the selectivity to CO_2_ results slightly increased by the K presence. However, the total NO_x_ reduction efficiency of the K/MgAlO_x_ catalysts is still not high enough; indeed, the best NO_x_ removal efficiency is no higher than 8%, which is not practically viable. The key reason is because MgAlO_x_ mixed oxides itself have relatively poor reductive activity.

Thus, in order to increase the total NO_x_ removal efficiency of these LDH-derived catalysts, a noble metal or a metal with redox properties similar to the noble metal is generally demanded. For these reasons, a catalytic system based on CuO has been considered by Wang et al. [25]. As expected, the ignition temperature (T_i_) is dependent on the soot–catalyst contact, being near 260 °C in tight contact conditions and 314 °C in loose contact; moreover, the maximum conversion of NO to N_2_ decreased from 40.4% to 29.2%, definitely higher than that on K/MgAlO_x_ catalysts. Wang et al. [26] have examined the catalytic property of Co–Al mixed oxides derived from hydrotalcites as a new active catalyst for the simultaneous NO_x_–soot removal reaction. Additionally, in this case, the catalytic activity was related to the redox properties of the catalyst affected by the Co content and calcination temperature. Indeed, by increasing the calcination temperature from 500 to 800 °C, both the activity of soot oxidation and the selectivity to N_2_ formation increased due to the enhancement of redox properties. The active species might come from Co_3_O_4_; indeed, when the Co spinel form is present, its reduction takes place in the same temperature window of soot oxidation suggesting that a redox-type mechanism acts for soot oxidation in the presence of O_2_/NO_x_ and that the catalyst is redox active, i.e., easily reducible and reoxidizable by gaseous oxidants. The occurrence of simultaneous NO_x_–soot removal reactions was confirmed by the presence of CO_2_, N_2_, and N_2_O between the reaction products: the oxidation of soot by either NO_x_ or O_2_ giving CO_2_, and the reduction of NO_x_ by soot to N_2_ and N_2_O. For the best formulation (i.e., with the Co/Al ratio of 5 and calcinations temperature of 800 °C) the ignition temperature of soot oxidation was 290 °C; however, the NO_x_ selective conversion to N_2_ remains too low, which is lower than 4%.

Ternary LDH-derived catalysts have also been proposed in this context. Li et al. [27] studied the hydrotalcite-based Mn_x_Mg_3__-x_AlO (Table 2) and found that the soot combustion activity follows the order: Mn_1.5_ > Mn_1.0_ > Mn_0.5_ = Mn_2.0_ > Mn_2.5_ = Mn_3.0_ > Mn-free, while the sample Mn_1.0_ shows the best performance in the simultaneous soot–NO_x_ removal. They conclude that the Mn^4+^ ions are the most active species for soot combustion and simultaneous soot - NO_x_ removal.

Later on, Li and their coworker [29] proposed a series of K-promoted hydrotalcite-derived Mn_1.5_Mg_1.5_AlO catalysts; the catalyst exhibits both an efficient soot oxidation and NO_x_ storage. Moreover, the authors suggest different pathway for the soot oxidation depending to the loading of potassium; in particular, when K is less than 10 wt %, the reaction follows the oxygen spillover mechanism, while for higher K content the direct participation of the surface nitrates in soot oxidation is claimed, hence suggesting the involvement of a redox mechanism occurring between nitrates and soot particles. Between all the surface species formed during the storage, DRIFT spectroscopy results revealed that potassium monodentate nitrate are the most reactive with soot and their formation is ruled by K loading. Moreover, a new phase was identified, i.e., K_2_Mn_4_O_8_ that proves to be highly active for soot combustion and NO_x_ reduction by soot.

A series of Mn-doped MgAlO_x_ was also prepared by Cui et al. [30]. The catalysts exhibited high NO_x_ storage capacity at low temperatures (150–300 °C), due to its greater surface area, improved reducibility and higher surface Mn^3+^ content. During the lean-rich cycling tests, the average NO_x_ removal rate can reach above 70% after Mn doping. However, the presence of soot has a slight detrimental effect on the NO_x_ uptake, that decreases from 426 µmol/g at 200 °C to 406 µmol/g. This could be related to the exothermic combustion of soot destabilized the stored species and the soot combustion produced CO_2_ would compete with NO_x_ for storage sites on the catalyst. Unfortunately, the authors do not investigate the activity of Mn-doped MgAlO_x_ directly in soot combustion.

**Figure 2:**
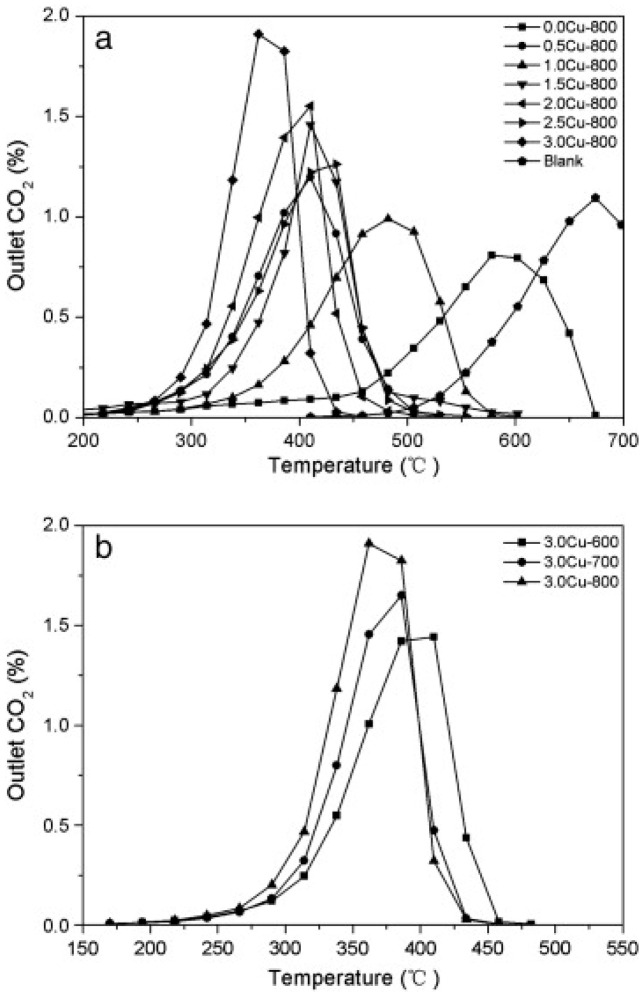
Catalytic performances for soot oxidation over CuMg/Al catalysts during NO_x_ soot reactions. Gas composition: NO 0.25 vol.%, O_2_ 5 vol.%, balance He; flow rate: 20 cm^3^/min Reproduced with permission from [28]. Copyright © 2012, Elsevier, Inc. (**a**)outlet CO_2_ concentration over CuMg/Al catalysts calcined at 800 °C at different Cu loading; (**b**) outlet CO_2_ concentration over 3.0 CuMg/Al catalysts calcined at different temperature.

Between the ternary LDH-derived catalysts, the effect of Co and Cu in the formulation has been also studied. Indeed, Cu- and Co-MgAl LDH-derived catalysts have demonstrated to be very active for the simultaneous catalytic removal of soot and NO_x_ due to their redox and acid–base properties. As a matter of fact, in the review of Yang et al. [9] some examples of CuMgAlO_x_ catalyst active in the simultaneous removal of soot and NO_x_ are reported. It has been verified that the catalytic activity strongly depends on the calcination temperature, being the optimal fixed at 800 °C [28,31]. Additionally, the results suggested that the addition of Cu significantly increased the activity of catalysts; among the tested catalysts, Cu(3)MgAlO_x_ sample calcined at 800 °C shows the best activity with T_i_ = 260 °C and total amount of N_2_ formed during the TPR run near 6.0 × 10^−5^ mol, as evident in Figure 2.

**Figure 3:**
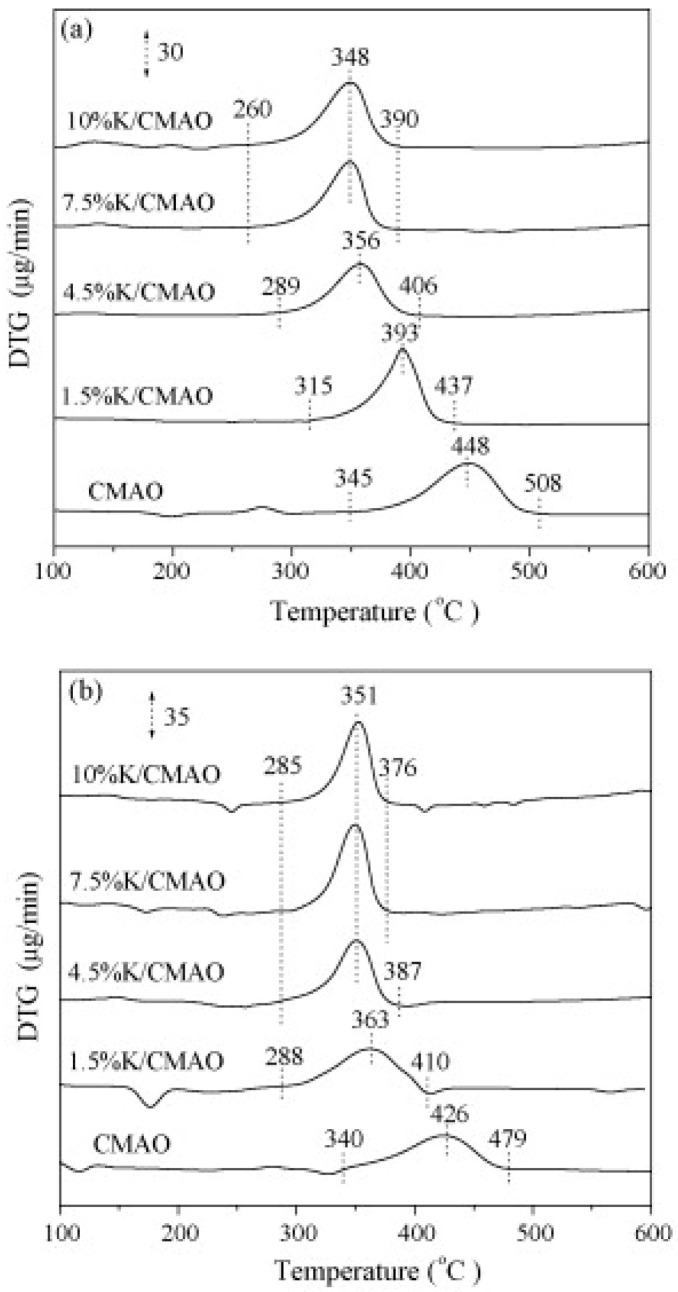
DTG profiles of soot combustion on x% K/Co-MgAlO catalysts (x = 0, 1.5, 4.5, 7.5 and 10) in different atmosphere: (**a**) air, (**b**) mixture of 400 ppm NO + 10% O_2_ balanced by N_2_. Adapted with permission from [13]. Copyright © 2009, Elsevier, Inc.

The effect of calcination temperature has been reported by Li et al. [12] for the Co_2.5_Mg_0.5_Al catalyst and 4.5% K-promoted sample, founding that simultaneous catalytic removal of soot and NO_x_ can be achieved over these catalysts in the temperature range of 300–700 °C. The catalyst 4.5%K/Co_2.5_Mg_0.5_Al calcined at 600 °C shows the best performance, not only for soot combustion but also for simultaneous soot–NO_x_ removal; in this case, the soot ignition temperature is near 330 °C and the NO_x_ reduction percentage results as high as 32%. This is attributed to its high surface K/Co atomic ratio and to the strong interaction between K and Co. To further study the performance of K-promoted Co_2.5_Mg_0.5_Al catalysts, the effects of K loading has been evaluated [13]. The results showed that the soot combustion activity is higher when K is present and it increases with K loading. Indeed, as it is reported in Figure 3, the onset temperature for soot oxidation shifts towards lower values upon increasing K loading.

The data reported in the Figure 3 clearly demonstrate that the soot oxidation activity of Co-MgAlO hydrotalcite is enhanced by the presence of K both in the air and in the presence of NO. Additionally, Zhang et al. [22] come to the same conclusions studying K/MgAlO catalysts. The authors attributed this positive effect of potassium to its strong interactions with Mg(Al) species; this weakens the bonds in CoAl_2_O_4_ spinel and Mg(Al)–O, facilitating the mobility of bulk lattice oxygen species. Li et al. [13] performed also NO_x_ storage experiments at 350 °C, i.e., the temperature at which the maximum conversion of soot is observed in the same conditions. The results showed that when Co-MgAlO catalysts are promoted with K, the NO_x_ storage capacity is increased (Table 3); moreover, the NO_x_ storage capacity increases with potassium loading. The NO adsorption is facilitated by activation of gaseous oxygen and NO itself due to the presence of electron-donating K species.

**Figure 4:**
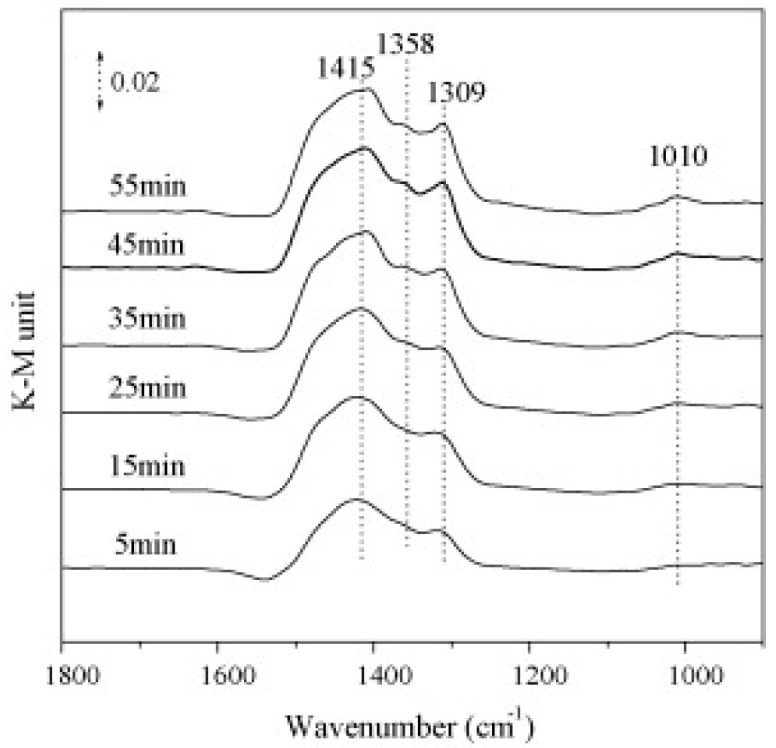
The in situ DRIFTS spectra of NO_x_ sorption on 1.5% K/Co-MgAlO catalyst. Adapted with permission from [13]. Copyright © 2009, Elsevier, Inc.

DRIFT studies reported in Figure 4 demonstrated that on the 1.5% K/Co-MgAlO catalyst the main adsorbed species are monodentate nitrates on K sites that have a stronger basicity than Mg sites. When potassium loading increases, monodentate nitrates transform into ionic species.

In summary, in this work the authors assumed a mechanism for NO_x_ adsorption over K/Co-MgAlO catalysts similar to that proposed for traditional K-based lean NO_x_ trap (LNT) catalysts like Pt/K/Al_2_O_3_ catalysts [32,33,34]. In fact, in these traditional catalytic systems Pt is the active site for NO oxidation, while K_2_O is assumed to be the main potassium species where the adsorption occurs; along the same lines, in K/Co-MgAlO catalysts the Co sites are thought to be responsible for the activation of NO molecules. It is important to note that although the NO_x_ storage capacity of K/Co-MgAlO catalysts is undoubted, nothing is reported about the reduction of stored NO_x_.

In the review by Yang et al. [9] the authors concluded that hydrotalcite-based catalysts have demonstrated to be active in the simultaneous removal of soot and NO_x_; indeed, in the K–M–O system new phase reactive oxygen species are present, which easily react with soot. These species, formed due to the strong interaction between K and metals on the support of catalysts, oxidized NO to NO_2_, being the first stored in the form of nitrates over K species while the former reacts with soot. Definitely, even if there is still room to improve both soot oxidation activity and the NO_x_ storage capacity of the LDH derived catalysts, in the future they could represent a viable solution for the vehicle emissions control systems.

### 2.2. Perovskite-Based Catalysts

Today, perovskites are considered as a viable alternative choice to PGM-based catalysts in the oxidation of particulate matter due to their ease of synthesis, high thermal stability and low cost compared to PGMs. In addition, these materials could be modified and doped with a wide range of elements in order to adapt their properties in relation to their specific applications [35,36,37].

The general chemical formula for perovskite compounds is ABO_3_, where “A” and “B” are two cations of very different sizes, and O is the anion that bonds to both. In general, “A” is rare earths (e.g., La, Ce, and Pr), alkali and alkaline earths (e.g., Cs, Sr, Ba, and Ca) larger than “B” transition metals (e.g., Co, Fe, Cu, Ni, Mn, Cr, and Al). A large number of metal ions having a different valence can replace both A and B ions, thus modulating the catalytic properties of these materials. Note that the catalytic properties of perovskite-type oxides basically depend on the nature of A and B ions; the A site ions are catalytically inactive, while catalytic activity is generally determined by the B cation. These materials have the capacity to form reactive oxygen species with high mobility, becoming a good option to replace noble metals for soot removal applications, like catalyzed DPF. Furthermore, alkali oxides like K or Li are often added to perovskites to improve soot combustion processes. Owing to their redox properties coupled to a high oxygen mobility, perovskites were used for soot oxidation also in the presence of NO, with particular attention to the NO oxidation reaction (being NO_2_ more reactive than NO in soot oxidation) and to the selectivity of the C + NO_2_ reaction. These studies are the basis for the development of perovskites catalysts for the simultaneous removal of soot and NO_x_ from automotive exhausts. In particular, the incorporation of dopants in the parent perovskite structure was indicated to improve both de-NO_x_ and de-soot catalytic activity. The application of perovskites catalysts in soot and NO_x_ control is summarized in Table 4.

A large number of investigations focused on mixed oxides with perovskite and spinel structures (i.e., see the review by Hernández-Giménez et al. [44] and references therein) aiming at developing efficient and cheap soot oxidation catalysts. Many catalytic formulations have been reported to promote soot combustion, including noble metals, alkaline metals and alkaline earth metals, transition metals that can accomplish redox cycles (e.g., V, Mn, Co, Cu, Fe, etc.), and internal transition metals (i.e., rare earth metals). In general, alkali metals are the most intensively studied dopants [45,46,47,48] even if, in some cases, potassium-containing catalysts are reported to suffer from low stability at high temperatures [49].

Mn-based materials are a good choice for oxidative applications. Teraoka et al. [38,45] first and Wang [50] later on, systematically investigated perovskite or spinel oxides for the simultaneous catalytic removal of soot and NO_x_ in oxygen-containing model exhausts, focusing the attention on the promoting effect of potassium. Indeed, doping with K can effectively reduce the ignition temperature of soot and improve the selectivity for reducing NO_x_ to N_2_. Both founded that nanosized La_1−x_K_x_MnO_3_ (x = 0.2, 0.25) exhibited a very high catalyzing activity under a loose contact conditions. The study of Teraoka [38] revealed that the La–K–Mn–O oxides are good candidates for the simultaneous NO_x_–soot removal reaction, as demonstrated by the formation of CO_2_ due to the oxidation of the soot and the reduction of NO into N_2_ (and N_2_O) observed in the same temperature range. The ignition temperature for soot oxidation decreases by increasing the K loading down to 208 °C when x = 0.4, while the 30% of reduced NO was converted to N_2_O. It is worth to note that the study has been performed under tight conditions between catalysts and soot particle. However, under practical conditions the contact between the catalysts on the surface of filter and PM particle is loose.

La–K–Mn–O systems have been considered also by Peron and Glisenti [39] in a very recent work, which focused on LaMnO_3_ compared with LaCrO_3_ as potential starting points for the substitution of noble metal catalysts. The perovskite formulation has been also modified by doping with K, and the resulting materials, i.e., La_0.8_K_0.2_CrO_3_ and La_0.8_K_0.2_MnO_3_ have been tested in soot oxidation by O_2_ and NO. Potassium has improved activity for both Cr- and Mn-containing perovskites, but Mn-containing perovskites results more active than chromites (T_max_ 314 °C vs. 337 °C).

The remarkable improvement in the soot oxidation activity also under the loose contact condition reported for chromites (LaCrO_3_) with the respect to manganite (LaMnO_3_), suggests that also other perovskite families, like cobaltites (LaCoO_3_), ferrites (LaFeO_3_), and titanites (SrTiO_3_) where La (or Sr) is partially substituted with K, could be proposed in this field. This is confirmed by Kureti et al. [51] reporting that iron-containing materials are promising catalysts for simultaneous NO_x_–soot removal. On the other hand, Fino et al. [52] investigated the effect of Cr/Fe presence, preparing a series of Cr- and Fe-substituted perovskite samples (LaMnO_3_, LaFeO_3_, LaCrO_3_, LaCr_0.9_O_3−δ_, and La_0.9_K_0.1_Cr_0.9_O_3−δ_) and testing them in the soot oxidation. The comparative analysis of such catalysts showed that chromite class are more active than LaMnO_3_ and LaFeO_3_ catalysts, having La_0.9_K_0.1_Cr_0.9_O_3−δ_ the lowest CO_2_ peak temperature (455 °C).

Cobaltites-based catalysts have been considered by Wang et al. [42] studying the catalytic performance of the La–K–Co–O perovskite oxide catalyst. The partial substitution of La^3+^ at A-site by alkali metal K^+^ enhanced the catalytic activity for the oxidation of soot particle and reduction of NO_x_. The results demonstrated that the La_0.70_K_0.30_CoO_3_ sample is a good candidate catalyst for the simultaneous removal of the soot particle and NO_x_; indeed, the combustion temperatures for soot particles are in the range from 289 to 461 °C, the selectivity of CO_2_ is very high near 98% and the conversion of NO to N_2_ is 34.6% under loose contact conditions. The authors proposed at least three possible mechanisms to explain the enhanced catalytic activity after K-doping: (i) the formation of high valance ion (Co^4+^) at B sites of perovskite, which had better catalytic oxidation activity than Co^3+^; (ii), the formation of oxygen vacancy, whose presence favors the NO adsorption; and (iii) the formation of oxide catalysts with nanometric size and thus the good contact between the catalysts and soot.

Recently, Dhal et al. [40] discuss the properties of nanometric La_1−x_K_x_MnO_3_, La_1−x_K_x_CoO_3_, La_1−x_K_x_FeO_3_, and La_1−x_Na_x_MnO_3_ perovskite-type oxide catalysts in the simultaneous removal of NO_x_ and soot. The reported results demonstrate that all of the catalysts were active in soot combustion, showing the manganite-based catalyst the lower ignition temperature (see Table 5). The removal of NO_x_ has been investigated in presence and absence of soot under cycling conditions, i.e., alternating lean-rich phases according to the DPNR concept (see below), and the NO_x_ conversion is reported in Table 5.

The authors founded that when K is not present in the formulation, the activity order for soot oxidation follows LaMnO_3_ > LaFeO_3_ > LaCoO_3_. On the other hand, the addition of K makes La_1−x_K_x_CoO_3_ more active than La_1−x_K_x_FeO_3_, while La_1−x_K_x_MnO_3_ remains the most active. They proposed a soot oxidation mechanism operating through different routes depending on the temperature: in the low temperature region, NO is initially oxidized to NO_2_ over catalysts, then NO_2_ oxidizes the soot particles; at high temperature besides the soot oxidation by NO_2_, the active oxygen on the surface of the catalysts can directly oxidize soot particles involving superficial oxide complexes intermediates. The N_2_ formation occurs through the reaction between the soot particles and adsorbed nitrate species acting as oxidant species.

Meanwhile, some studies have reported that the partial substitution of lanthanum by strontium in LaCoO_3_ and LaMnO_3_ perovskites improved NO-to-NO_2_ oxidation activity beyond that of the model Pt-based catalyst formulation [53,54]; furthermore, other studies have demonstrated that strontium substitution of lanthanum could modulate the NO_x_-to-N_2_ efficiency of perovskite-based catalysts [55,56]. Indeed, it has been found that both La_1−x_Sr_x_CoO_3_ and La_1−x_Sr_x_MnO_3_ perovskites compared favorably with a commercial platinum-based catalyst in DOC (diesel oxidation catalyst) and LNT (lean NO_x_ trap) systems [57], with improved activity in soot oxidation [58,59].

Additionally, the dual substitution of Ag and K in place of La in LaMnO_3_ has been demonstrated giving improved activity in the simultaneous soot–NO_x_ reaction [41]. As a matter of fact, soot combustion is largely accelerated, with the temperature for maximal soot conversion (T_m_) lowered by at least 50 °C; moreover, the silver substitution at A site of perovskite increases the NO_x_ reduction efficiency (Table 6). Indeed, the metallic Ag can efficiently adsorb NO and O_2_, and oxidize NO to NO_2_ and, given that NO_2_ is a better oxidizer for soot than NO or O_2_, it enhances the activity for both soot oxidation and NO_x_ reduction. From the data reported in the work, it appears evident that the co-presence of Ag and K at the A-site of LaMnO_3_ catalyst improved the NO conversion from 45% to 64%, better than in the only Ag-substituted catalyst (53.28%), even the temperature for maximum NO conversion was higher for K-substituted catalyst than the only Ag-substituted catalyst.

Another example is reported by Li and coworkers [60], who prepared a series of nanometric Fe-substituted La0_.9_K_0.1_Co_1−x_Fe_x_O_3−δ_ (x = 0, 0.05, 0.1, 0.2, and 0.3) perovskite catalysts for diesel soot oxidation, NO_x_ storage, and simultaneous NO_x_–soot removal. The reported results showed that the formulation with x = 0.1 is the most active in the removal of both soot and NO_x_; in fact, the maximal combustion rate temperature is reached at 362 °C, the storage capacity is 213 µmol/g and the NO_x_ reduction by soot is 12.5%. The reason of such higher activity is related to the high oxidation capacity of Fe, so NO is efficiently oxidized to NO_2_ that is adsorbed on the catalyst surface more efficiently than NO. However, also in this work the reduction of stored NO_x_ is reported in term of NO_x_ released under the programming temperature.

Considering the titanites family, the activity and stability of two potassium-perovskite catalysts (K/SrTiO_3_ and Sr_0.8_K_0.2_TiO_3_) and a potassium-copper perovskite catalyst (K–Cu/SrTiO_3_) for soot combustion in the NO_x_/O_2_ gas mixture has been analyzed by López-Suárez and coworker [43]. Even the authors do not report any data on NO conversion, all the catalysts result in being active in the soot combustion in NO_x_/O_2_ mixtures because of a decrease in the onset temperature for soot combustion and an increase significantly in the soot combustion rate. However, all the potassium-containing catalysts suffer deactivation; the most significant decrease in catalyst activity takes place between the first and the second TPR (temperature programmed reduction) cycle, being the deactivation in further cycles much less important; indeed, the T_50%_ (temperature required to achieve the 50% soot conversion) values increase by more than 150 °C from the first to the second cycle (i.e., from near 450 °C to ca. 600 °C).

**Figure 5:**
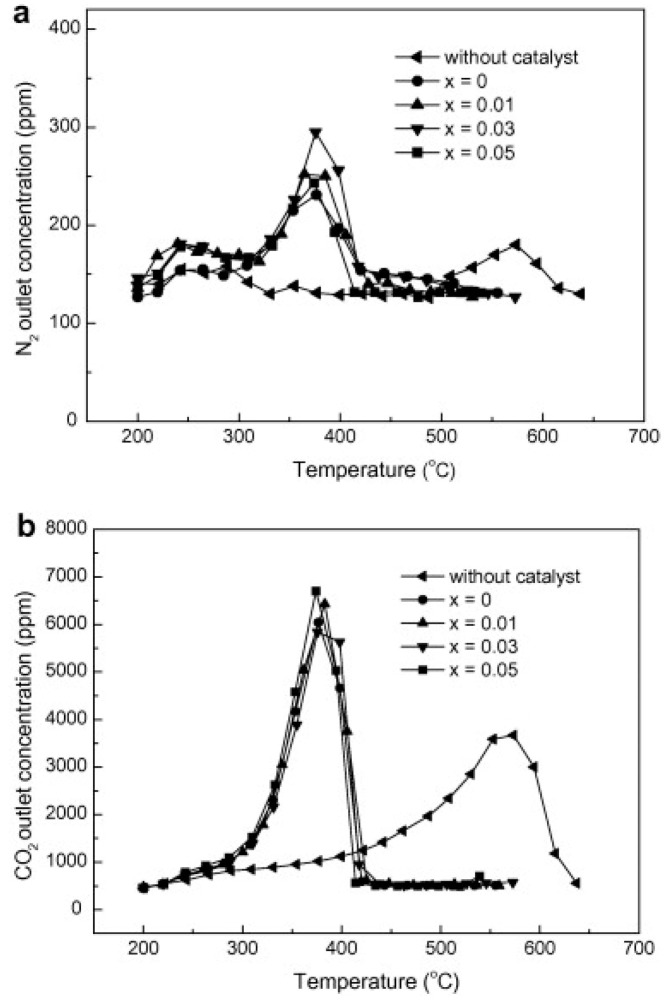
The outlet N_2_ and CO_2_ concentration profiles during temperature programmed reaction over La_1−x_Ce_x_NiO_3_ catalysts (0 ≤ x ≤ 0.05). Substitution degrees (x) are indicated within the figure. (**a**) Outlet N_2_ concentration profiles and (**b**) outlet CO_2_ concentration profiles. Reprinted with permission from [61]. Copyright © 2009, Elsevier, Inc.

Other more complex formulations have been proposed in the literature. Zhao et al. [61] studied La_1−x_Ce_x_NiO_3_ (0 ≤ x ≤ 0.05) perovskite catalysts for simultaneous removal of nitrogen oxides and diesel soot. Indeed, the results reported in Figure 5 indicate that N_2_ and CO_2_ are produced almost at the same temperature range thus evidencing the occurrence of the simultaneous removal of NO_x_ and soot. In particular, compared to the non-catalytic combustion of soot under the same reaction conditions, the ignition temperature decreases from 450 to 300 °C. The partial replacement of Ce for La increases the concentration of Ni^2+^ thus promoting the catalytic activities. The redox properties of the Ce^3+^/Ce ^4+^ couple and the capacity of cerium oxide to exchange oxygen with the gas phase are also behind the good catalytic performance of ceria-based materials as soot combustion catalysts [62].

Very recently, simultaneous NO_x_ storage and soot oxidation performances of doped barium cerate perovskite materials have been reported by Maffei et al. [63]. The NO_x_ storage capacity of the co-doped (Zr and Co) barium cerate was found comparable to traditional Pt-based lean NO_x_ traps, i.e., 228 µmol/g at 380 °C and in the presence of water; moreover, the lowest T_max_ for soot oxidation resulted near 436 °C. However, in this case the rich step typical for DPNR systems has not been analyzed.

It is undoubted that the soot–NO_x_-catalyst system implies a very complex interplay and that the chemistry of the involved reactions is also complex. For these reasons, how occurs the simultaneous removal of NO_x_ and soot under the condition of rich oxygen over perovskite-type catalysts remains almost unclear and several mechanisms have been proposed. A scheme of the complex pathway of the reactions involved in the simultaneous removal of soot and NO_x_ has been proposed by Liu et al. [64] in the case of La_2−x_Rb_x_CuO_4−__λ_ perovskite-like oxide catalysts.

**Figure 6:**
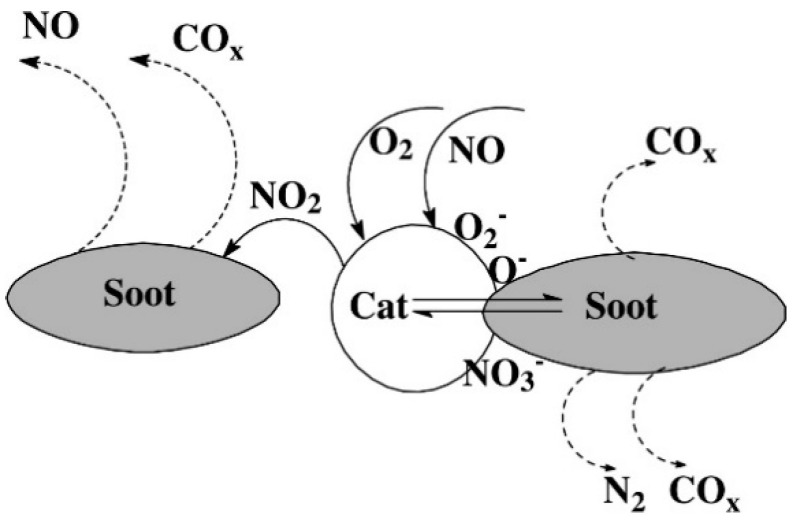
The scheme of the reaction mechanism for the simultaneous removal of soot and NO_x_ over La_2x_Rb_x_CuO_4λ_ perovskite-like oxide catalysts. Reprinted with permission from [64]. Copyright © 2008, American Chemical Society.

This scheme (Figure 6) supposes the formation of O_2_^−^ and O^−^ species by dissociative adsorption of O_2_ on the catalyst surface. When the contact between catalyst and soot is guaranteed, they react to each other forming CO and CO_2_, otherwise the catalyst cannot promote the soot oxidation reaction. Nevertheless, NO can be oxidized to NO_2_ according to reaction (1), and since this species is more reactive towards soot than NO and/or O_2_, the reaction between NO_2_ and soot occurs also under loose contact conditions according to global stoichiometry of reactions (2) and (3):

2 NO + O_2_ → 2 NO_2_ (1)

C + 2 NO_2_ → CO_2_ + 2 NO (2)

C + NO_2_ → CO + NO (3)

Additionally, on the catalyst surface nitrate species could be formed, according to reactions (4) and (5), which in turns could be reduced by soot giving nitrogen reactions (6) and (7):

NO + O_2_^−^ → NO_3_^−^ (4)

NO_2_ + O^−^ → NO_3_^−^ (5)

2 NO_3_^−^ + 2 C → N_2_ + 2 CO_2_ + 2 O^−^ (6)

2 NO_3_^−^ + 4 C → N_2_ + 4 CO + 2 O^−^ (7)

Despite the extensive research efforts, the activities of the perovskite catalysts remain typically inferior to those of the precious metal-based counterparts, being the role of noble metal mainly to oxidize NO to NO_2_, which subsequently oxidizes soot to CO and CO_2_. Therefore, NO_2_ is used as an intermediate to facilitate an indirect contact between the catalyst and soot. For this reason, a small amount of noble metal like Pt or Pd has been incorporated into the B site of the perovskite structure. It has been reported that the catalytic activity is remarkably improved; in particular, Pd results more effective than Pt for NO_x_ reduction at lower temperatures and the Pd–K interaction to promote the reduction of pre-adsorbed NO_x_ [65,66].

To conclude, it is worth to note that the concept of “simultaneous removal of soot and NO_x_” in most cases is intended as oxidation of soot and simultaneous reduction of NO_x_, i.e., the oxidation of soot occurs in the presence of NO_x_, which in turn are reduced to N_2_. This is very different from the meaning given by the DPNR Toyota concept, as detailed in the following. Indeed, in the literature rarely the perovskites behaviors are analyzed in the complete lean-rich cycles typical of DPNR catalysts. The main problem is that the perovskite-like structure is extremely damaged under a reducing atmosphere, but it seems to be recovered by calcining at 400 °C under lean conditions. Furthermore, the effect of H_2_O or CO_2_ normally present in the exhausts often is not considered [35].

## 3. PGM-Based Catalysts for Simultaneous Removal of Soot and NO_x_

Noble metal-based catalysts have been extensively studied in the catalytic soot combustion in order to enhance the intrinsic redox ability of catalysts. Although many methods are employed to synthesize supported noble metal nanoparticles, including impregnation, ion exchange, liquid-phase chemical reduction, and co-precipitation, there is much interest in novel methods to synthesize improved catalysts. Indeed, it is well-known that the high surface-to-volume ratio of the particles is crucial for the activity of the metal nanoparticle. So, the ability to synthesize stable and precisely engineered nanoparticles in order to optimize their catalytic activity is highly desired. The atomic layer deposition (ALD) technique can offer several benefits when compared to conventional nanoparticle synthesis methods, for example extreme film thickness uniformity, precise thickness control, excellent step coverage, and high reproducibility. The thickness of the films can be easily controlled by controlling the number of deposition cycles. For these reasons, ALD can be used to deposit catalytic coatings on high surface area porous powder supports or on geometrically complex structures [67,68,69] such as particulate filters in diesel engine exhaust systems.

Metal-doped hydrotalcites have been proposed due to the ability of LDHs to improve the dispersion of precious metals [66]. Indeed, as already discussed above, K-supported MgAlO (K/MgAlO) systems showed improved NO_x_ adsorption at high temperatures [18]; in addition the optimization with noble metals (i.e., mainly Pt and Pd) improved the catalytic performance, particularly at low temperatures [70]. Moreover, it has been found that there is an interaction between the hydrotalcite-like derivative structure doped with Pd and the matrix oxide [71], so that Pd promotes the reduction of the composite oxide and increase active oxygen species in the surface, as well. For these reasons, the soot–NO_x_ simultaneous removal performance of the hydrotalcite-like catalyst was improved by combining K and Pd, which improve the catalytic soot combustion activity and the NO_x_ storage/reduction efficiency, respectively.

**Figure 7:**
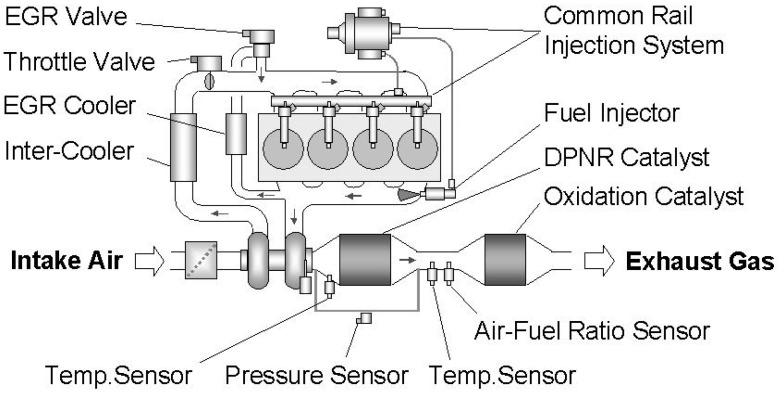
Engine system configuration with the diesel particulate–NO_x_ reduction (DPNR) system.

Among the others PGM-based catalytic systems, the best known is the already mentioned DPNR system patented by Toyota group in the early 2000s [72,73,74,75]. This system applies a NO_x_ storage and reduction catalyst (like LNT catalyst) uniformly coated on the wall surface and in the fine pores of a highly porous filter substrate. The direct injection diesel engine configuration includes a newly-developed fuel injector installed on the upper stream of catalyst for adding fuel to the exhaust system, a common-rail fuel injection system capable of carrying out high-pressure, high-precision fuel injection control and an electrically controlled EGR system (Figure 7), in order to make it possible to drive with a rich air-fuel ratio, and to achieve precious catalyst temperature control.

**Figure 8:**
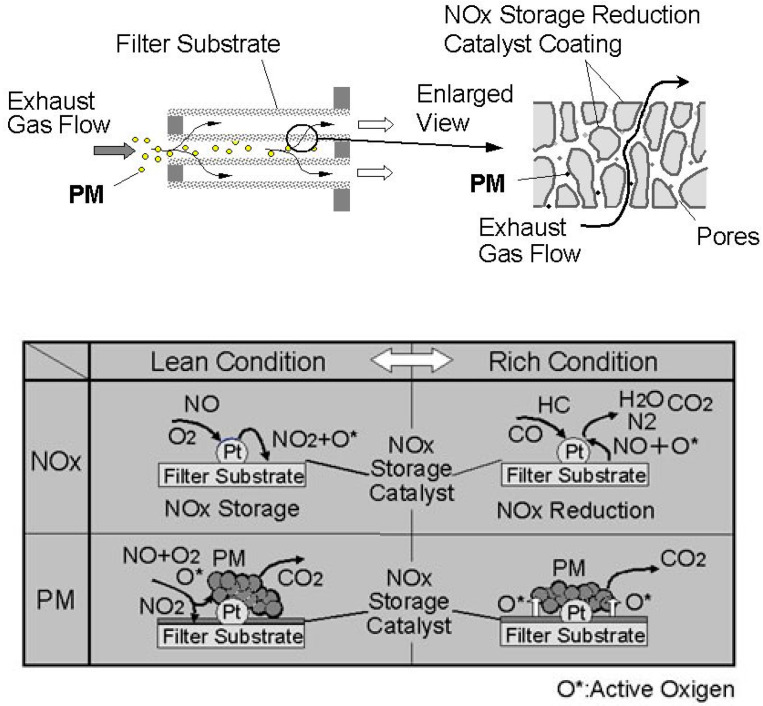
DPNR catalyst cross-section and NO_x_ and particulate matter (PM) treatment mechanism. Reprinted with permission from [76]. Copyright © 2003, Springer Nature.

NO_x_ are adsorbed on the DPNR catalyst during the lean phase and are reduced during the subsequent rich phase. Particulate matter emitted from the engine is trapped by the filter substrate and then, starting from 300 °C, it is continuously oxidized by the oxygen and NO_x_ contained in the exhaust gas and by an active form of oxygen produced on the catalyst by repeated switching between a lean air-fuel ratio and rich air-fuel ratio (Figure 8). Both pore structure of the filter substrate and the catalyst are specifically optimized to improve both soot trapping and oxidation efficiency.

The DPNR catalyst is formally a NSR (NO_x_ storage reduction) catalyst used in the lean NO_x_ trap (LNT) system. It is constituted by three key components, i.e., a high surface area support (e.g., γ-alumina), a noble metal (Pt), and an alkaline or alkaline-earth metal oxide; the catalyst presents a high NO_x_ storage capacity combined with a high soot oxidation capability.

In this section a survey on the existing literature on the DPNR concept is presented based on both the traditional Pt-based systems and the new Pt-free ones.

### 3.1. DPNR Catalysts for the Simultaneous Removal of NO_x_ and Soot

In the last decade, the DPNR technology catalysts have attracted increasing attention due to the well performances of LNT catalysts also in the soot combustion. Among the others, Castoldi et al. [77], extensively studied the DPNR strategy based on a traditional LNT formulations, i.e., Pt-Ba-based catalysts supported on alumina. As a matter of fact, the Pt-Ba/Al_2_O_3_ catalyst/soot mixture (9:1 w/w) has been tested at constant temperature under cycling conditions, i.e., alternating rectangular step feeds of NO/O_2_ (lean condition) with rectangular step feeds of H_2_ (rich condition).

**Figure 9:**
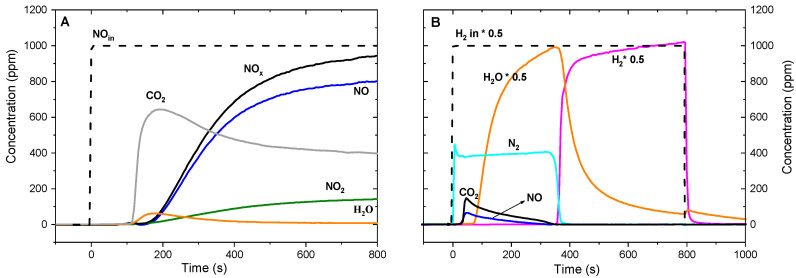
Lean and rich sequence (**A**, **B** respectively) obtained at 350 °C in the case of a Pt–Ba/Al_2_O_3_/soot mixture. Adapted with permission from [77]. Copyright © 2006, Elsevier, Inc.

A typical example of lean-rich sequence is reported in Figure 9. During the storage phase (Figure 9A), NO_x_ are efficiently stored over the Pt-Ba/Al_2_O_3_ in the presence of soot with formation of surface nitrates, according to the global reaction (8):

2 BaO + 4 NO + 3 O_2_ → 2 Ba(NO_3_)_2_ (8)

In the meantime, CO_2_ evolution is also observed and ascribed to soot oxidation. It is worth noting that CO_2_ is observed late (near 150 s) compared to the beginning of the storage phase due to its adsorption over barium sites with formation of BaCO_3_, and not correlated to a delayed combustion. In fact, when the same lean phase was performed in the presence of CO_2_, CO_2_ formation was immediately observed after NO addition to the reactor.

The promoting effect of NO on soot combustion over Pt-based catalysts was ascribed to the ability of Pt to catalyze the oxidation of NO into NO_2_ (i.e., a stronger oxidant than oxygen), which favors the soot combustion according to the following overall reactions, already proposed in the literature:

NO + ½ O_2_ → NO_2_

2 NO_2_ + C → CO_2_ + 2 NO

Indeed, the amount of NO_2_ detected results lower than that measured without soot at the same temperature, suggesting its consumption due to the reaction with soot.

It is noteworthy that the NO originated from reaction (2) can be oxidized again to NO_2_ according to reaction (1), resulting in the so-called NO recycle, which is expected to speed up the soot combustion [78]. Moreover, to explain the positive effect of NO_2_ formation on soot combustion a cooperative NO_2_/O_2_ oxidation mechanism was also invoked. On this regard, several C-O_2_-NO_2_- reaction mechanisms were proposed. Indeed, some authors suggest the occurrence of a reaction between O_2_ and the surface intermediates species arising from the interaction between soot and NO_2_ [79,80]; others report the existence of two parallel routes: (i) direct soot oxidation by NO_2_ and (ii) soot combustion involving cooperatively both O_2_ and NO_2_, strongly catalyzed by Pt [81,82,83,84]. The latter favors the decomposition of surface oxygen species originated by O_2_ and reactions that involve species adsorbed onto the catalyst surface.

Furthermore, it has been suggested that another contribute to soot combustion is given by stored nitrates. In line with this hypothesis Castoldi et al. compared the behavior of Pt-Ba/Al_2_O_3_ and Pt/Al_2_O_3_ catalysts. They found that the Ba-free sample efficiently oxidizes soot but cannot store NO_x_. On the other side the Pt-Ba/Al_2_O_3_ sample exhibited a similar soot oxidation capacity despite the occurrence of NO_x_ storage on the catalyst surface (i.e., resulting in lower gas phase NO_x_ concentration than the Ba-free sample) suggesting specific oxidizing properties of the surface NO_x_ species. Accordingly, as it will be detailed later, the following global reaction could be hypothesized:

C + 2 NO_3_^−^ → CO_3_^2=^ + 2 NO + ½ O_2_ (9)

During the reduction of stored NO_X_ with H_2_ (Figure 9B), N_2_ production was observed (along with minor amounts of NO) accordingly to the overall stoichiometry:

Ba(NO_3_)_2_ + 5H_2_ → BaO + N_2_ + 5H_2_O (10)

The water formed during reduction displaced carbonates previously formed and this accounts for the evolution of CO_2_ during the reduction. Moreover, this CO_2_ could also derive from limited soot combustion during the rich phase. In fact, according to the literature the NO_x_ reduction involves at first the release of NO_x_, which then are reduced to nitrogen over Pt [2]. Accordingly, the released NO_x_ may oxidize soot leading to the formation of CO_2_. The oxidation of soot during rich pulses is also supported by Sullivan [85] and by Toyota researchers that, by means of ESR (electron spin resonance) experiments, correlated the higher soot oxidation rate observed in the presence of rich pulses to the formation of so-called activated oxygen species (e.g., superoxide species) generated under rich conditions [72].

**Figure 10:**
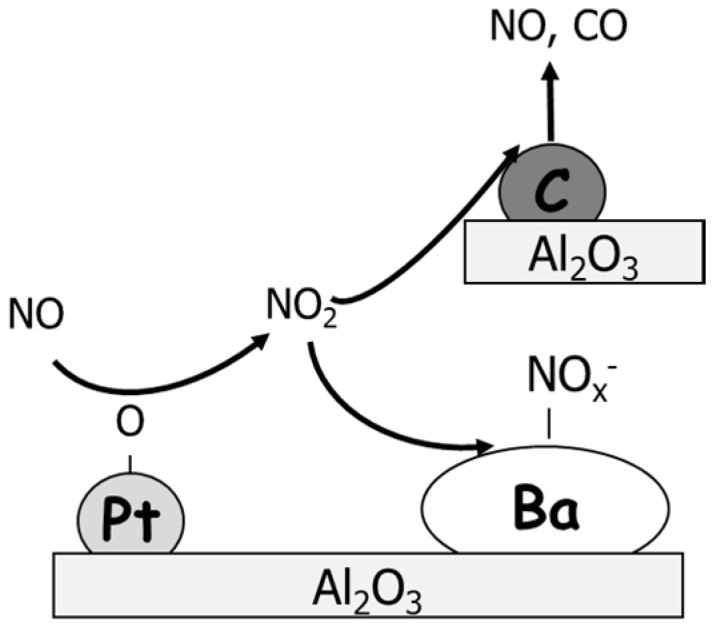
Proposed mechanism by which particulate matter decreases the NO_x_ adsorption capacity of a NO_x_ trap.

The investigation has been extended by Lietti and coworkers considering the effect of temperature, the presence of CO_2_/H_2_O, and the NO inlet concentration on both NO_x_ storage reduction activity and soot oxidation capacity of model LNT catalyst [86,87,88]. As pointed out by the authors, the presence of soot strongly influences the storage behavior of NO_x_ over Pt-Ba/Al_2_O_3_, decreasing its storage capacity in the range 250–350 °C regardless of the NO inlet concentration used in the experiment (i.e., 500 ppm and 1000 ppm). However, considering sequential lean-rich cycles, it appeared that the residual soot loading directly affects the performance of Pt-Ba/Al_2_O_3_. In fact, both the NO_x_ breakthrough and the amounts of NO_x_ stored at the steady state were found to progressively increase during the lean-rich sequence, i.e., upon decreasing the soot loading as consequence of soot combustion. Similar trends were also reported by Cortés-Reyes et al. [89] who observed lower NO_x_ adsorption rate in the presence of soot than in the absence (from 9.5 × 10^−3^ to 4.5 × 10^−3^ mg min^−1^), while once the catalyst becomes regenerated the adsorption rate of NO_x_ backs to its original values. This negative effect of soot on the NO_x_ storage capacity was explained by a less availability of NO_2_ for storage due to its involvement in the soot oxidation, i.e., Ba and soot compete in the reaction with NO_2_ [85,90]. On the basis of the so-called “nitrate” route for the storage of NO_x_ (i.e., NO oxidation to NO_2_ and subsequent NO_2_ adsorption in the form of nitrates via a disproportion reaction [91]), soot was blamed to offer another path for the use of NO_2_ rather than the NO_x_ storage process, i.e., being NO_2_ involved in the soot oxidation instead of surface NO_x_ formation (Figure 10). Accordingly, in the presence of soot lower amounts of NO_2_ were measured during the lean phase than that observed in the absence of soot.

**Figure 11:**
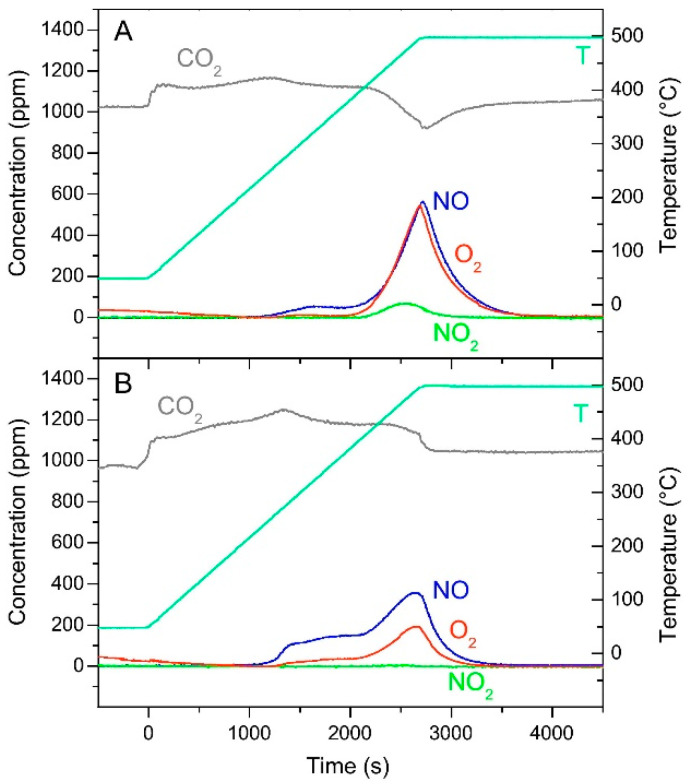
TPD (Temperature Programmed Decomposition) run after NO_x_ adsorption at 350 °C over (**A**) Pt-Ba/Al_2_O_3_ catalyst and (**B**) Pt-Ba/Al_2_O_3_/soot mixture. Reprinted with permission from [87]. Copyright © 2011, Elsevier, Inc.

On the other side, the direct interaction between surface nitrates and soot particles via a surface reaction was reported to positively affect the soot combustion process. In particular, the direct participation of the surface NO_x_ in soot combustion was suggested to occur without the preliminary thermal decomposition of stored species. In fact, specific studies on the thermal stability of stored NO_x_ species [86,87,88] confirmed that in the absence of soot the thermal decomposition of stored species occurs near the adsorption temperature (in this case 350 °C, Figure 11A) and according to the global stoichiometry of reactions (11) and (12). Note that an uptake of CO_2_ was observed due to the formation of barium carbonates.

Ba(NO_3_)_2_ → BaO + 2 NO + 3/2 O_2_ (11)

Ba(NO_3_)_2_ → BaO + 2 NO_2_ + 1/2 O_2_ (12)

On the other hand, in the presence of soot (Figure 11B) the decomposition is observed at a lower temperature; moreover, also the products distribution (i.e., amount of NO, O_2_, and NO_2_) is different, and the registered concentrations obey to the global stoichiometry of reaction between soot and nitrates adsorbed species (reaction (9)), already suggested.

The above results on one side confirmed the destabilizing effect of soot on the stored NO_x_ and on the other supported the active role of the surface NO_x_ in soot combustion, without the necessity of their prior thermal decomposition. As a matter of fact, the stored nitrates were found to oxidize soot at temperatures lower than those of their decomposition without soot. The direct reaction between stored NO_x_ and soot was explained invoking the mobility of surface nitrates in analogy with the reduction mechanism proposed for LNT catalysts [92,93]. Since in the literature it has been suggested that nitrates adsorbed on Ba are mobile in the presence of reducing centers (i.e., reduced Pt sites kept in the reduced state by the reductant), similarly the presence of soot (a reductant) was supposed to be the driving force for the mobility of the nitrates, which may ultimately oxidize soot.

With this in mind, NO_x_ surface species act as an oxidant toward soot, which itself function as a reducing center; this redox mechanism represents a pathway parallel to the traditional NO_2_–soot oxidation occurring in the presence of gas-phase NO_2_ during the DPNR operations.

The occurrence of a direct surface reaction between carbon particles and NO_x_ stored species was also proposed by Tschamber and coworkers [94,95,96,97] to explain the decrease in the NO_x_ storage activity when soot is present. The proximity between the storage sites, Pt sites, and their contact with soot is a key factor in the NO_x_ storage behavior of the catalysts in the presence of soot according to the two possible pathways for NO_x_ storage from NO/O_2_ mixtures proposed by the literature [91], i.e., the “nitrate” and “nitrite” route, respectively. The ‘‘nitrite’’ route involves the stepwise oxidation of NO resulting to the formation of surface nitrites, and a cooperative interaction between Pt and Ba nearby sites is suggested as crucial for this route, which hence implies the existence of a strong Pt–Ba interaction. Nitrites are then gradually oxidized into nitrates, which prevail at catalyst saturation. Assuming that the proximity of Pt decreases the reducing character of soot via formation of C(O) oxygen surface complexes and consequently the redox interaction occurring between soot and nitrates, the authors suggested that nitrate species formed close to Pt (via “nitrite” route) are less destabilized by soot. Besides, the nitrate species formed far from Pt (via “nitrate route”) were indicated to suffer much more from contact with soot according to the above proposed surface reaction. As a matter of fact, TEM analysis revealed the occurrence of structure modifications following carbon combustion, i.e., platinum sintering and Ba agglomeration. These structural modifications were suggested to decrease the proximity between the platinum and storage sites, resulting in a decrease in the NO_x_ storage capacity though the “nitrite route”. Additionally, the effect of water was investigated and a non-cumulative effect of carbon and H_2_O on the NO_x_ storage capacity was pointed out resulting from the competition between the destabilization of the weakly bonded surface nitrate, by carbon, and the enhancement of bulk nitrates formation, by water.

**Figure 12:**
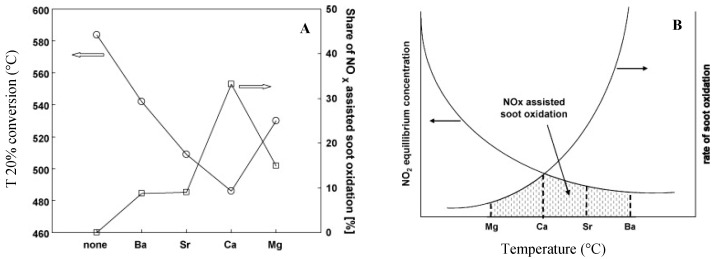
(**A**) Influence of alkali-earth cations on T20% conversion and share of NO_x_ assisted soot oxidation and (**B**) schematic explanation of NO_x_ assisted soot oxidation for different alkali-earth metals. Reprinted with permission from [101]. Copyright © 2009, Elsevier, Inc.

The importance of the interaction between surface nitrates and soot and the beneficial effect on the soot oxidation process is also documented by Sullivan et al. [98] suggesting that on the Na/Al_2_O_3_ catalyst NO adsorbs in the form of nitrites/nitrates, which can further decompose, i.e., releasing NO_2_. Shuang et al. [99] indicated the NO_2_ derived from nitrates decomposition on Pt-Mg/Al_2_O_3_ catalysts as beneficial for the soot oxidation activity. Krishna and Makkee [100] claimed the involvement of surface nitrates in soot oxidation via the release of NO_2_ in the gas phase studying soot oxidation with the NO/O_2_ mixture over Pt–K/Al_2_O_3_ and Pt–Ba/Al_2_O_3_ LNT catalysts. In another work, Kustov and Makkee [101] analyzed the impact of stored nitrates on soot combustion over Al_2_O_3_ supported alkali-earth oxides (i.e., Ba, Sr, Ca, and Mg), founding that stored nitrates promote to the soot oxidation by decreasing the temperature of soot combustion up to almost 100 °C, as it clearly appears from Figure 12A where the activity of given catalysts is reported as a function of the temperature of 20% soot conversion.

Moreover, it was found that the temperature of the nitrates decomposition and the temperature of soot oxidation by NO_2_ are crucial for an efficient soot oxidation (see Figure 12B). Indeed, the efficiency of NO_x_ utilization depends on both these factors, being the soot oxidation by NO_2_ limited by the kinetics of the NO_2_-C reaction and by the low thermodynamic stability of NO_2_ at high temperatures.

Sanchez and coworkers showed the direct participation of the surface nitrates in soot oxidation, without the need of their preliminary thermal decomposition, over K-containing lanthanum supported catalysts [102,103]. Other authors support the same thesis over Cs-loaded MnO_x_–CeO_2_ catalysts [104], and over BaAl_2_O_4_ catalysts [105,106].

As already discussed above, the interaction between soot and nitrates (or, in general, NO_x_ stored species) influences the oxidation of soot and it is extremely efficient when K is present in the catalyst formulation. This beneficial effect was ascribed to the formation of K-based low melting point/volatile compounds, which can favor the surface mobility of the active species and consequently the soot–catalyst contact, that is essential for soot oxidation [107,108]. Moreover, a synergistic effect occurring between K and Pt has been reported to increase the mobility of actives species formed over the potassium thus enhancing the soot oxidation activity [81,100]. Many authors investigated the performances of K-based LNT catalysts in the soot combustion simultaneously to the NO_x_ storage. Among others, Matarrese et al. [33,34,109,110] investigated the effect of the substitution of Ba by K in LNT catalysts. They founded a significantly higher activity for soot combustion for the Pt-K/Al_2_O_3_ catalyst compared to that of Pt-Ba/Al_2_O_3_ sample, while the catalysts showed a similar de-NO_x_ activity. The high soot oxidation activity of Pt-K/Al_2_O_3_ was explained taking into account the high mobility of K-nitrates, likely enhancing the soot–catalyst contact and hence boost in the soot combustion, according to the literature already reported. This was confirmed also by dedicated TPD (temperature programmed desorption) and TPO (temperature programmed oxidation) experiments [111] which pointed out the lower thermal stability of the NO_x_ species stored over K and the higher reactivity of K-nitrates towards soot then Ba-ones. TPO results performed in the presence of oxygen by M. Cortés-Reyes et al. [82] suggests the following reactivity order in terms of onset temperature for soot oxidation.

Pt-K/Al_2_O_3_ > K/Al_2_O_3_ > Pt/Al_2_O_3_ = Pt-Ba/Al_2_O_3_ = Ba/Al_2_O_3_ = Al_2_O_3_.

These results were in agreement with those of Krishna and Makkee [100] who reported that both K/Al_2_O_3_ and Pt–K/Al_2_O_3_ show a superior soot oxidation capacity than Pt/Al_2_O_3_ and Pt-Ba/Al_2_O_3_.

The main drawback of the K-based catalyst was the partial deactivation upon ageing with repeated NO_x_ storage reduction cycles in the presence of soot, showing a decreased NO_x_ storage capacity and also a lower soot oxidation activity. This was correlated to a reduced availability of the K actives species, due to partial loss of K and/or to participation of the K actives species in interactions with alumina and/or Pt [34].

### 3.2. Pt-Free DPNR Systems

Well-known PGM catalysts are expensive and because of PGM low abundance, they may undergo increasing prices upon increasing demand [112,113]. For this reason, in the last years great efforts have been made to develop low-cost PGM-free catalysts, also for DPNR applications.

Castoldi et al. [114] proposed silver catalysts supported on Al_2_O_3_, CeO_2_, and ZrO_2_ and containing Ba or Sr as storage components. The results showed all the investigated catalysts in NO/O_2_ promote the soot combustion at low temperatures (i.e., ca. 250 °C). Such a good oxidation activity was likely related to the ability of metallic silver to form suboxide species and/or superoxide O_2_^-^ ions, which are expected to assist carbon oxidation by O_2_. Besides, all the catalysts were active in the oxidation of NO to NO_2_ so that the presence of silver was indicated to promote also the NO_2_-assisted soot oxidation. All the Ag-containing catalysts were able to remove NO_x_ both in the absence and in the presence of soot, being able to adsorb them in lean conditions and subsequently reduce in rich ones. A lower NO_x_ storage capacity was observed for the Sr-based samples if compared to the Ba-based samples, which was ascribed to the different basicity of strontium and barium. The reactivity of a model LNT Pt-Ba/Al_2_O_3_ system was also considered for comparison purposes. The results showed that on one side, without soot, the storage capacity of the Ag-systems outperforms that of a traditional Pt–Ba/Al_2_O_3_ LNT catalyst; on the other side, soot negatively affects the storage capacity of Ag-based catalysts more than model LNT catalysts. Besides the Ag-systems, particularly those supported on ceria, were found much more active in the simultaneous soot combustion than the Pt-based catalyst. This was correlated to the generation and participation of oxygen active species from silver and/or ceria. In conclusion, Ag-based catalysts were proven as a promising alternative to Pt-based catalysts for the simultaneous removal of soot and NO_x_ even if their NO_x_ reduction performances should be further improved, being low the selectivity to nitrogen.

Matarrese et al. [115] investigated a second class of Pt-free catalysts based on ruthenium supported on different supports (Ce_0.8_Zr_0.2_O_2_, ZrO_2_, and Al_2_O_3_) with Ba or K as NO_x_ storage materials. According to TPO experiments, all the investigated systems were found more active in soot combustion than traditional Pt-based LNT materials. The presence of well dispersed ruthenium nanoparticles was suggested to promote the dissociative adsorption of oxygen leading to the formation of active oxygen species, which eventually can be transferred to the carbon promoting its oxidation. In particular, K-containing catalysts exhibited very low onset temperature in the presence of NO/O_2_ (i.e., in the range 220–235 °C) pointing out a synergistic interaction between Ru and K. In addition, all the formulations were able to accomplish the simultaneous removal of soot and NO_x_, under isothermal cycling conditions, especially, NO_x_ storage performances similar to conventional Pt-based catalysts were reported. Beside, K catalysts exhibited higher de-NOx and de-soot activity than Ba catalysts. However, also the NO_x_ reduction efficiency of the Ru-containing catalysts requires further improvements.

Castoldi et al. compared Ag-, Ru-, and Pt-based catalysts supported on Al_2_O_3_ and containing Ba as NO_x_ storage material [116]. The results confirmed the superior soot oxidation activity for Ag-Ba/Al_2_O_3_ and even more for Ru-Ba/Al_2_O_3_ than model Pt-Ba/Al_2_O_3_ LNT catalysts. Besides their NO_x_ storage capacity was comparable to that of traditional Pt LNT catalysts. However, also in this case, the N_2_ selectivity during reduction was rather low, particularly in the case of Ag-Ba/Al_2_O_3_ for which NO was the main reduction product (i.e., N_2_ selectivity near 30%). Of note, as opposed to model Pt-based catalysts, the NO_x_ storage capacity of Ru-Ba/Al_2_O_3_ was not negatively affected by soot; moreover, in the case of the Ru-Ba/Al_2_O_3_ catalyst very similar NO/NO_2_ ratios were measured with and without soot. This was correlated to the lower involvement of NO_2_ in the soot oxidation (in line with the direct involvement of active oxygen species formed by metallic Ru) or to the higher oxidation efficiency of NO to NO_2_.

Since ceria-based oxides are recognized among the most promising materials for soot combustion [62,117], ceria/zirconia (CZ)-based catalysts, doped with Pt, Au, Ru, or Fe and containing K, have been considered for both soot oxidation and simultaneous removal of NO_x_ by Matarrese et al. [118]. The results pointed out that all the CZ formulations and in particular the Ru-containing catalysts are able to decrease the soot ignition temperature in the presence of only oxygen at temperatures below 300 °C. Moreover, when operating under isothermal cycling conditions the Ru-based catalysts were found much more active than Pt-K/Al_2_O_3_ in both soot combustion and NO_x_ storage capacity. The high NO_x_ storage activity was explained basing on FT-IR experiments. In fact, the initial formation of nitrites, which evolve fast to nitrates, was observed on both Ru and Pt-systems. Moreover, the contribution of also bulk nitrates and mono-nitrosyl species on Ru was found for the Ru system. However, also in this case, the Ru-based catalysts showed a poor NO_x_ reduction activity if compared to model LNT catalysts.

Of note, it should be remembered that Ru-based catalysts have often been accused of low stability due to the possible loss of active phase (i.e., with consequent catalyst deactivation) via volatilization of Ru oxides. In particular, several recent studies [119,120] reported that at high temperatures (i.e., higher than 700 °C) RuO_2_ is oxidized into volatile RuO_4_. Notably, focusing on this crucial aspect, Villani et al. [121] investigated the stability of Ru/Zeolite based catalysts and found 900 °C as the upper limit temperature for practical applications of ruthenium catalysts, while Matarrese et al. [118] performed repeated TPO soot oxidation cycles reporting a quite stable and reproducible behavior.

Bueno-Lopez et al. [122] investigated Cu/Ce_0.8_M_0.2_O_δ_ catalysts (M = Zr, La, Ce, Pr, or Nd) for the simultaneous removal of soot and NO_x_ and, also in this case, the presence of soot affected the NO_x_ storage activity depending on the different nature of the acid/basic character of the doping metal, resulting in being detrimental for catalysts with very basic supports (e.g., doped with La). This was explained with the possible competition for the adsorption sites between the CO_2_ emitted during soot combustion and NO_x_, in agreement with the DRIFT results. The behavior of the Pr-based catalyst (i.e., the best formulation for both NO_x_ adsorption and soot oxidation) was further investigated at 400 °C under lean-rich cyclic conditions. N_2_ was reported as the main reduction product under rich condition. Besides, soot oxidation was reported during H_2_ pulses, which was tentatively explained invoking (i) with the destabilization of the stored nitrates upon H_2_ admission leading to the formation of NO*_2_* that may oxidize soot and/or (ii) with the localized increase in temperature due to the oxidation of H_2_.

More recently copper/ceria-based catalysts were investigated for soot oxidation by Giménez-Mañogil et al. [123] who pointed out the synergistic interaction between copper and cerium, as responsible for the catalytic performances. In particular, copper in close contact with ceria was indicated to improve the catalysts reducibility, which was found to play a key role in their catalytic behavior towards NO oxidation to NO_2_ and soot combustion processes.

## 4. SCR/DPF Combined Technologies for the Simultaneous Removal of Soot and NO_x_

Among the combined technologies proposed to reduce simultaneously soot and NO_x_ and realize more compact systems, an important role is played by SCR catalysts coated on a particulate filter, i.e., the so-called SCRF^®^ systems (SCRF^®^ is a registered trademark of Johnson Matthey Public Limited Company. All rights reserved) [124,125].

There are some difficulties in combining de-NO_x_ and de-soot functionalities in terms of technology related to the combination of the de-NO_x_ and PM abatement functionalities, mainly to the interactions between the SCR and soot chemistries that can result in a SCRF performance lower than that of the individual devices. Additionally, the impact on the mass-transfer characteristics by the presence of soot can affect in a negative way the de-NO_x_ efficiency. In order to minimize the impacts of these factors, the catalytic material and the coating process are very important. Moreover, since it is desirable to introduce the highest possible quantity of catalyst in the pores of the filter, the porosity of the latter must be carefully considered due to the limits established by the maximum pressure loss a filter component can have. In addition, it is necessary to prevent thermal damage of the SCR-coating and the DPF-monolith.

A very crucial aspect to consider is the analysis of the performance of the SCRF^®^ in comparison to that of the individual SCR and DPF devices. In particular, the close interplays between the SCR and DPF functions for the general case of soot-loaded devices deserve attention [126,127,128]. For example, it should be considered the involvement of NO_2_ in both SCR reactions and passive soot oxidation but also the right compromise between de-NO_x_ performance, filtration efficiency, and pressure drop behavior when selecting the optimal washcoat amount. Moreover, the influence of soot presence on NO_x_ conversion must be taken into considerations. In fact, the reaction of NO_2_ with soot forming NO affects the in situ NO_2_/NO_x_ ratio, which as it is known directly impacts on the de-NO_x_ performance. Therefore, a promoting or a detrimental effect takes place depending on the operating conditions [126]. Finally, also the thermal stability of SCR catalyst formulations needs attention bearing in mind the severe thermal conditions occurring within filters [129].

In 2012, Kröcher and a coworker [130] investigated the SCR reaction in the presence of NH_3_ over soot (i.e., both model and real diesel soot), in the temperature range between 200 and 350 °C. They observed SCR activity on diesel soot, which could be exploited to improve future diesel exhaust after treatment systems, where both DPF and SCR systems have to be used in series to reach the desired emission limits. The authors proposed a mechanism for NO_x_ reduction over diesel soot where the first reaction step is the disproportionation of NO_2_, which is followed by the formation of ammonium nitrates and nitrites.

2 NO_2_ + H_2_O → HONO + HNO_3_ (13)

HONO and HNO_3_ are assumed to remain physisorbed on the surface, where they form ammonium nitrate (NH_4_NO_3_) and ammonium nitrite (NH_4_NO_2_) in the presence of NH_3_:

2 NH_3_ + HONO + HNO_3_ → NH_4_NO_3_ + NH_4_NO_2_ (14)

**Figure 13:**
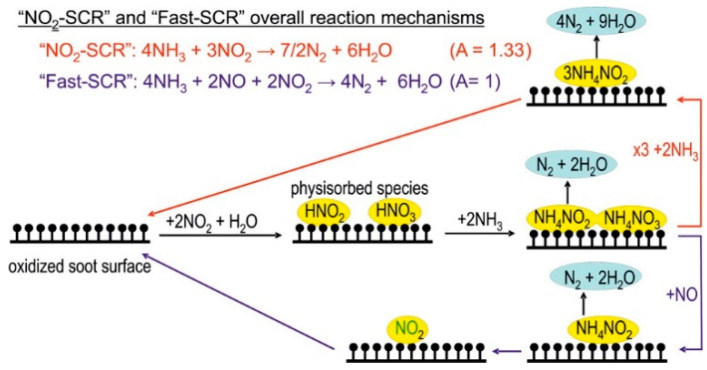
Suggested mechanism for the selective catalytic reduction of NO_x_ on diesel soot. The dark points symbolize acidic carbon surface species supporting NH_3_ adsorption. Reprinted with permission from [130]. Copyright © 2012, American Chemical Society.

The nitrites decompose directly into N_2_ and H_2_O (indeed NH_4_NO_2_ is unstable at temperatures above 60 °C), whereas the nitrates have to be reduced to nitrites either by NO or in its absence by NH_3_, which also leads to the formation of N_2_ and H_2_O. The SCR chemistry on the soot surface is summarized schematically in Figure 13.

Regarding the SCR component, the use of Cu- or Fe-based ion-exchanged zeolite for SCRF^®^ applications [131,132,133,134,135,136] requires specific attention, i.e., in achieving the high NO_x_ conversion over a wide temperature range, avoiding NH_3_-slip together with high efficiency for soot filtering and removal, and avoiding thermal damage to both SCR coating and SCRF^®^-monolith [137]. However, high NO_x_ conversion and high soot-filtration efficiency cannot often be accomplished simultaneously. Indeed, as reported by different authors [138,139,140], a soot-loaded SCRF^®^, operated in the cake filtration regime, showed less NO conversion than a soot-free SCRF^®^.

**Figure 14:**
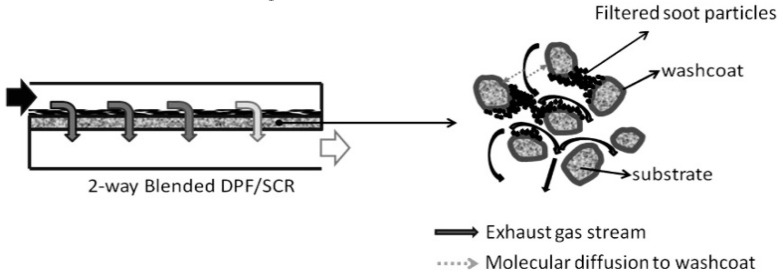
Schematic diagram of blended 2-way DPF/selective catalytic reduction (SCR). Reprinted with permission from [141]. Copyright © 2012, American Chemical Society.

It is worth noting that the combination of DPF and SCR functionalities into a single device may result in problems, which are absent in separate devices; indeed, the operation of the 2-way DPF/SCR device (Figure 14) might be different from that of conventional DPF and SCR, and the fuel penalty during filter regeneration must be taking into account.

Park et al. [141] considered a Cu−zeolite catalyst coated inside the DPF substrate material. The results showed a soot ignition temperature near 300 °C, i.e., considerably lower than thermal soot oxidation so that copper zeolite was indicated as a promising catalytic material not only for SCR reactions but also for soot oxidation. In fact, the soot deposited in the form of deep-bed filtration contacts the catalytic material directly and it starts to oxidize first upon increasing the temperature. On the other hand, the authors founded that the de-NO_x_ reactions are influenced by the deposited soot. First, the de-NO_x_ reaction is hindered by the deposited soot due to the resistance for mass transfer from the exhaust gas stream to the catalytic sites; second, significant amounts of NO_2_ in the soot cake layer are consumed by the soot oxidation reactions. In the absence of soot, the de-NO_x_ performance becomes poor when the NO_2_/NO_x_ ratio exceeds 0.5. However, when soot is fully loaded in the 2-way device and at temperatures suitable for the NO_2_-assisted soot oxidation (e.g., 400 °C), the de-NO_x_ performance is almost independent of the NO_2_/NO_x_ ratio. It is clear from the obtained results that to completely describe the phenomena occurring in a DPF/SCR system, it is necessary to develop and implement new kinetic models taking into account both soot filtration and NO_x_ reduction by SCR. For this purpose, SCR kinetics (see Table 7) was incorporated in the model describing the transport and reaction phenomena inside a wall-flow type substrate, while for soot filtration and oxidation, mathematical formulations based on a soot cake layer filtration model and both NO_2_- and O_2_-based soot oxidation reactions are incorporated [142,143].

Watling et al. [127] reported the development, validation, and application of a one-dimensional model for an SCRF^®^. The model described in that paper was developed by combining kinetics for either a Cu-zeolite or an Fe-zeolite SCR catalyst, originally developed for a flow-through monolith, with a physical model for a coated DPF. It has been demonstrated that this model is capable of predicting NO_x_ conversion and NH_3_ slip from an SCRF^®^ system in a real diesel exhaust. Since the design and control of the SCRF^®^ strongly depends on the interaction between NO_x_ reduction and soot oxidation reactions taking place in close vicinity, this model has been also applied to investigate the interaction between SCR and DPF functionality. The presence of soot on the SCRF^®^ is predicted to have no significant impact on NO_x_ conversion, while SCR activity (NO_x_ reduction) is predicted to significantly retard the rate of soot removal by oxidation with NO_2_. Indeed, NO_x_ reduction by SCR occurs much more rapidly than the soot–NO_2_ reaction.

Furthermore, Tronconi et al. [144] studied the interactions between soot and Cu/zeolite powder, with particular attention to the chemistries of NH_3_-SCR and of soot combustion at the lab-scale level. All the experimental tests have been performed over physical mixtures consisting of powdered Cu-SCRF and soot, thus emphasizing the interactions between the two reacting systems. The addition of NH_3_ was found to greatly reduce the low-temperature combustion of soot by NO_2_, i.e., the actual oxidizing agent of soot at low temperatures. In fact, given that NO_2_ is converted in the NH_3_-SCR reactions (i.e., fast SCR and NO_2_ SCR reactions), a substantial decrease in the passive soot regeneration by NO_2_ can be expected in SCRF systems. On the other hand, the impact of soot on the SCR reactions (i.e., NH_3_ oxidation, standard SCR and fast SCR activities) was marginal. Besides the SCR activity in excess of NO_2_ was promoted because of the low-temperature interaction between NO_2_ and soot, which leads to a more favorable NO_2_/NO ratio, closer to the optimal 1/1 molar ratio.

Similar results were found for instance by Schrade et al. [128] and Mihai et al. [145], who observed only a slight decrease of the NO_x_ conversion (up to 5%) for the standard-SCR reaction in the presence of engine soot on the SCRF (loaded on an engine test bench). Cavataio et al. [140] also reported a decrease in NO_x_ conversion in the standard- and fast-SCR for a soot-loaded SCRF, but on a much larger extent (up to 20%, using model-soot from a soot generator). In both case, these results were explained by blocking of the catalytically active sites.

Along the same lines, Lopez et al. [146] studied vanadia-SCR catalyst coating combined with a wall flow particulate filter. The results confirmed that significant NO_x_ and PM reduction could be obtained over transient cycles and at steady state conditions, being more than 70% NO_x_ conversion over a degreened vanadia-SCR/DPF. Passive filter regeneration was also investigated, obtaining both a good passive regeneration and a good particle number (PN) filtration.

**Figure 15:**
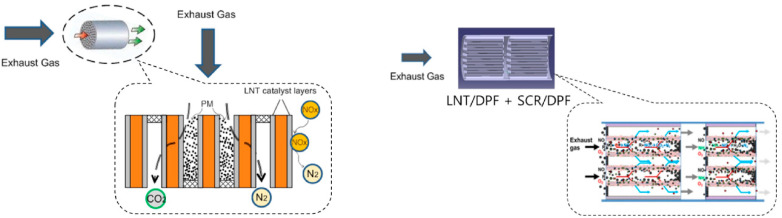
Fundamental principles of emission reduction in after-treatment systems: (**A**) lean NO_x_ trap (LNT)/DPF system and (**B**) LNT/DPF + SCR/DPF hybrid system. Adapted with permission from [125]. Copyright © 2018, Elsevier, Inc.

Very recently, Martinovic et al. [147,148] investigate the integration of soot oxidation and NO_x_ SCR by a two-component selective catalytic system and the interaction between them. The SCR catalysts were either Fe- or Cu-ZSM-5, while as the soot oxidation catalyst, CeO_2_-PrO_2_ (namely CP) was impregnated with K (called KCP). The authors found that physically mixing the commercial SCR catalyst with the soot oxidation one, it is possible to significantly decrease the onset temperature for soot combustion and simultaneously increase the NO_x_ conversion; indeed, NO is oxidized to NO_2_, which participates in the fast SCR reaction. Moreover, in this physical mixture, the soot was oxidized mainly by O_2_, since the contribution of NO_2_ was limited because it reacted in the SCR reaction (kinetically much faster). Interestingly, the authors conclude that their results have been obtained at the laboratory scale with the main aim of providing a detailed study on the interaction between a soot oxidation catalyst and a SCR catalyst. Thus, on a real monolith not only chemical interactions, but also fluid-dynamics and pressure drop, catalyst loading and distribution in the monolith, contact between soot and catalyst, contact length with the filtered soot cake represent key parameters to be taken into account. Finally, also coupled LNT-SCR/DPF systems have been also considered given that LNT catalysts can be significantly improved through the addition of a downstream SCR catalyst [149,150]. Moreover, to solve some problems typical of each of these after-treatment units, Kang et al. [125], introduce a LNT/DPF+SCR/DPF hybrid system (Figure 15). In a previous work, Choi and Lee [151] investigated the LNT/CDPF catalyst system for simultaneous removal of NO_x_ and soot (Figure 15A), where the LNT/DPF approach closely resembles the DPNR concept. LNT(2Pt20Ba)/CDPF coated with cordierite substrate showed the highest de-NO_x_ performance among other LNT catalysts. Moreover, the addition of 5% Co improved both the NO_x_ conversion performance and also the PM oxidation rate that resulted higher if compared to bare DPF, which was not coated with the LNT catalyst. Subsequently, Kang et al. [125] introduced a hybrid system of LNT/DPF+SCR/DPF (Figure 15B). The results showed that the NO_x_ conversion of the hybrid system was 40% compared to 25% of the LNT/DPF system. Moreover, the PM oxidation activity in the hybrid system was higher than in all the other configurations (i.e., hybrid system > LNT/DPF > bare DPF > SCR/DPF). The conclusions of their work are very interesting, being the de-NO_x_ and de-PM activities of the hybrid system superior to that of the single LNT/DPF system. Indeed, NO_2_ and NH_3_ forming in LNT/DPF under a rich air-to-fuel ratio are used as a reductant for the SCR/DPF catalyst of the hybrid system LNT/DPF+SCR/DPF. In addition, the SCR/DPF increased the NO_x_ conversion through HC-SCR [125].

## 5. Conclusions

The need to decrease NO_x_ and soot emissions is still critical and it is an unsolved challenge as indicated by the continuous increase of stringent regulation limits for both NO_x_ and diesel soot. Indeed, the EURO VI levels are limited to 0.08 g/km for NO_x_ and 0.005 g/km for soot. For these reasons, research efforts are strongly focusing on effective techniques to meet the EURO VI standards in general. To do this, the automotive industry has been forced to propose after-treatment solutions often based on several catalytic converters and in addition resulting in rather high-pressure drops, sophisticated control technologies, high cost, considerable weight, and space consumption, as well. Therefore, the simultaneous removal of soot and NO_x_ in a single catalyzed device represents a viable solution, also in view of the advantages that can be obtained in terms of both investment costs and pressure drop reduction.

This review focused on papers dealing with catalytic materials proposed and tested in the simultaneous catalytic removal of NO_x_ and soot, most of them based on laboratory studies. One of them is represented by hydrotalcite-derived mixed metal oxides. At the laboratory scale, hydrotalcite-based catalysts including potassium and cobalt in their formulations gave promising results in both soot combustion and NO_x_ storage in the same temperature range (200–400 °C). With regard to NO_x_ storage activity, K-promoted catalysts showed high trapping efficiency due to the formation of several kinds of N-containing surface species. Additionally, perovskite catalysts have been found as efficient catalysts for simultaneous abatement of diesel soot and nitrogen oxides resulting in performance close to those of PGM catalysts. However, in a short time it is more likely the simultaneous use of perovskites and noble metals rather than the total replacement of noble metals by perovskite by exploiting their specific advantages. Moreover, for both two categories of catalysts mainly the ability to store NO_x_ is considered and discussed.

Model Pt-based LNT catalysts are able to simultaneously remove NO_x_ and soot when operated under cycling conditions, i.e., alternating lean-rich phases according to the DPNR strategy. For these catalytic systems the simultaneous removal is really effective. Indeed, these systems are able to store NO_x_ under lean conditions and subsequently reduce them under rich ones even in the presence of soot while soot combustion simultaneously occurs. The soot oxidation is more efficient during the storage phase than during the rich one. Pt promotes soot combustion by catalyzing the oxidation of NO into NO_2_ by O_2_, the most effective oxidation agent being NO_2_. Additionally, the nature of the NO_x_ storage component (i.e., alkaline or alkaline earth) directly affects soot oxidation. In particular K-based system show higher performances in the soot oxidation if compared to Ba-based because of the high mobility of the active K surface species (i.e., low melting point/volatile compounds, eutectics with other catalyst components, etc.), which can improve the contact between catalyst and soot and consequently the soot oxidation activity. Besides the nature of the Pt-O-alkaline/alkaline earth interaction determines the temperature range in which the combustion process is effective. However, K-based systems are blamed for low thermal stability, which is associated to several technological problems, particularly the loss of active phase with consequent catalysts deactivation.

The current trend is looking for novel Pt-free catalytic formulations aiming at lowering the cost of the DPNR technology. In particular, ceria-based catalysts doped with Ag, Ru, or Cu should be considered as a promising alternative to Pt-based catalysts for the simultaneous removal of soot and NO_x_ as a result of higher soot oxidation activity and lower detrimental effect of soot on the amounts of stored NO_x_, as well. However, in most cases their reactivity in the reduction of the stored NO_x_ should be further improved towards N_2_ selectivity.

## Figures and Tables

**Table 1:** Literature review of hydrotalcite-based catalysts for the simultaneous removal of soot and NO_x_.

Hydrotalcites Families	Catalysts	Preparation Method	Reaction Conditions	Ref.
Binary MgAlO_x_ LDH	A series of K-supported MgAl with different amount of K doping (2, 5, 8 and 15 wt.% of K)	Co-precipitation; K addition by impregnation; calc. 600 °C	Cat/soot = 9/1; 9.97% O_2_ + He or 1050 ppm NO + He; total flow 100 mL/min; heating rate 5 or 10 °C/min	[22,23,24]
	CuAlO_x_ catalyst	Co-precipitation; calc. 800 °C	Cat/soot = 9/1; 0.25% NO + 5.0% O_2_ + He; total flow 20 cm^3^/min; heating rate 1.6 °C/min	[25]
	CoAlO_x_ catalyst	Co-precipitation; calc. 500 °C e 800 °C	Cat/soot = 20/1; 0.25 vol.% NO + 5 vol.% O_2_ + He, total flow 80 cm^3^/min, heating rate 1.4 °C/min	[26]
Ternary MgAlO_x_ LDH	A series of Mn-doped MgAl with different amount of Mn doping (from 0.5 to 3)	Co-precipitation; calc. 800 °C	Cat/soot = 20/1; 750 ppm NO + 10 vol.% O_2_ + N_2_, total flow 240 cm^3^/min, heating rate 10 °C/min	[27]
	A series of Cu_x_Mg_3-x_Al with different Cu contents (0.5, 1.0, 1.5, 2.0, 2.5, 3.0)	Co-precipitation; calc. 600 °C–700 °C–800 °C	Cat/soot = 20/1; 0.25 vol.% NO + 5 vol.% O_2_ + He; total flow 20 cm^3^/min, heating rate of 1.6 °C/min.	[28]
	Co_2.5_Mg_0.5_Al	Co-precipitation; calc. 500 °C, 600 °C, 700 °C or 800 °C	Cat/soot = 20/1; 400 ppm NO + 10 vol.% O_2_ + N_2_; total flow 400 mL/min; heating rate of 10 °C/min.	[12,13]
Quaternary MgAlO_x_ LDH	A series of K-doped Co_2.5_MgAl with different amount of K doping (1.5, 4.5, 7.5, 10)	Co-precipitation; K addition by impregnation; calc. 600 °C	Cat/soot = 20/1; 400 ppm NO + 10% O_2_ + N_2_; total flow 20 mL/min	[13]
	A series of K-doped Mn_1.5_MgAl with different amount of K doping (x = 1.5, 4.5, 7.5, 10, 15, 20)	Co-precipitation; K addition by impregnation; calc. 800 °C	Cat/soot = 20/1; 750 ppm NO + 10 vol.% O_2_ + N_2_, total flow 240 cm^3^/min, heating rate 10 °C/min	[29]

**Table 2:** NO_x_ storage capacities and NO_x_ reduction percentages of the Mn_x_Mg_3__-x_AlO catalysts. Adapted with permission from [27]. Copyright (2010) American Chemical Society.

Sample	NO_x_ Uptake (µmol/g_cat_)	NO_x_ Reduction Percentage (%)
Mn-free	373 (100–272 °C)	7.2 (278–700 °C)
Mn0.5	657 (100–404 °C)	20.4 (327–614 °C)
Mn1.0	502 (100–386 °C)	24.0 (295–554 °C)
Mn1.5	271 (100–336 °C)	12.6 (308–700 °C)
Mn2.0	233 (100–355 °C)	6.9 (253–460 °C)
Mn2.5	85 (100–202 °C)	6.5 (263–618 °C)
Mn3.0	108 (100–213 °C)	10.4 (212–648 °C)

**Table 3:** NO_x_ storage capacities over Co-MgAlO and the K-promoted catalysts. Adapted with permission from [13]. Copyright © 2009, Elsevier, Inc.

Sample	NO_x_ Uptake (mg/g_cat_)
Co-MgAlO	24.10
1.5% K/Co-MgAlO	31.75
4.5% K/Co-MgAlO	51.88
7.5% K/Co-MgAlO	56.03
10% K/Co-MgAlO	61.24

**Table 4:** Literature review of perovskite-based catalysts for the simultaneous removal of soot and NO_x_.

Perovskites Families	Chemical Composition	Catalysts	Preparation Method	Reaction Conditions	Ref.
Manganites	A_1–x_ R_x_MnO_3_	La_1−x_K_x_MnO_3_ (x = 0.1, 0.2, 0.25, 0.3)	Co-precipitation; calc. 850–950 °C	Cat/soot = 20/1; NO (0.5%) + O_2_ (5%) + He; total flow 20 cm^3^/min; heating rate 1 °C/min	[38]
		La_1−x_K_x_MnO_3_	Citrate-combustion route; calc. 750 °C	Cat/soot = 10/1; 10% O_2_ + 0.5% NO + He; total flow 100 mL/min; heating rate 1 °C/min	[39]
		La_1−x_K_x_MnO_3_	Complex combustion method; calc. 800 °C	Cat/soot = 10/1; NO 1000 ppm + O_2_ 0.5 vol% + He. total flow 70 mL/min; heating rate 5 °C/min.	[40]
		La_0.7_Ag_0.3_MnO_3_	Solid-state method; calc. 600 °C	Cat/soot = 20/1; 10% NO-Ar mixture 30 mL/min, oxygen 10 mL/min, air 60 mL/min; heating rate 10 °C/min	[41]
Cobaltites	A_1−x_R_x_CoO_3_	La_1−x_K_x_CoO_3_	Citric acid-ligated method; calc. 800 °C	Cat/soot = 5/1; 5% O_2_ + 2000 ppm NO + He; total flow 50 cm^3^/min; heating rate 2 °C/min	[42]
		La_1−x_K_x_CoO_3_	Complex combustion method; calc. 800 °C	Cat/soot = 10/1; NO 1000 ppm + O_2_ 0.5 vol% + He. total flow 70 mL/min; heating rate 5 °C/min.	[40]
Ferrites	A_1−x_R_x_FeO_3_	La_1−x_K_x_FeO_3_	complex combustion method; calc. 800 °C	Cat/soot = 10/1; NO 1000 ppm + O_2_ 0.5 vol% + He. total flow 70 mL/min; heating rate 5 °C/min.	[40]
Chromites	A_1−x_R_x_CrO_3_	La_1−x_K_x_CrO_3_	citrate-combustion route; calc. 700 °C	Cat/soot = 10/1; 10% O_2_ + 0.5% NO + He; total flow 100 mL/min; heating rate 1 °C/min	[39]
Titanites	A_1−x_R_x_TiO_3_	Sr_0.8_K_0.2_TiO_3_	sol–gel method; calc. 850 °C	Cat/soot = 4/1; 500 ppm NOx + O_2_ 5% + N_2_; heating rate 10 °C/min.	[43]

**Table 5:** Ignition temperature (T_i_) of soot combustion and NO_x_ conversion and of La-based catalysts. Adapted with permission from [40]. Copyright © 2017, Elsevier, Inc.

Catalyst	T_i_	NO_x_ Conversion
LaCoO_3_	300 °C	45% @400 °C
La_1−x_K_x_CoO_3_	200 °C	50% @305 °C
La_1−x_K_x_MnO_3_	150 °C	53% @300 °C
La_1−x_K_x_FeO_3_	250 °C	48% @320 °C

**Table 6:** Different catalysts’ activity. Reprinted with permission from [41]. Copyright © 2018, Bulletin of Chemical Reaction Engineering and Catalysis.

	Soot Oxidation		NO Reduction	
Catalyst	T_i_ (°C)	T_m_ (°C)	T_max_-NO (°C)	Conversion (%)
LaMnO_3_	205	380	386	45.3
La_0.65_Ag_0.35_MnO_3_	152	300	301	53.2
La_0.65_Ag_0.1_K_0.25_MnO_3_	135	355	368	64.4

**Table 7:** SCR reaction and reaction rate used by Park et al. [141,143].

Description	Reaction	Reaction Rate
NH_3_ adsorption	NH_3_ + S → NH_3_-S	$R_{1}=A_{1}C_{NH}{}_{3}\left(1-\theta \right)$
NH_3_ desorption	NH_3_-S → NH_3_ + S	$R_{2}=A_{2}exp\left(\frac{E_{a,2}}{RT}\right)\theta$
NH_3_ oxidation	2 NH_3_-S +3/2 O_2_ → N_2_ + 3 H_2_O + 2 S	$R_{3}=A_{3}exp\left(\frac{E_{a,3}}{RT}\right)C_{O_{2}}\theta$
NH_3_ oxidation	NO + 1/2 O_2_ → NO_2_	$R_{4}=A_{4}exp\left(\frac{E_{a,4}}{RT}\right)C_{NO}C_{O_{2}}^{1/2}-k_{4,b}C_{NO_{2}}$ where k4,b=A4exp(Ea,4RTKeq)CNOθ $K_{eq}=exp\left(-\frac{\Delta S}{R}\right)exp\left(-\frac{\Delta H}{RT}\right)$
Standard NH_3_ SCR	4 NH_3_-S + 4 NO + O_2_ → 4 N_2_ + 6 H_2_O + 4 S	$R_{5}=A_{5}exp\left(\frac{E_{a,5}}{RT}\right)C_{NO}\theta$
Rapid NH_3_ SCR	2 NH_3_-S + NO + NO_2_ → 2 N_2_ + 3 H_2_O + 2 S	$R_{6}=A_{6}exp\left(\frac{E_{a,6}}{RT}\right)C_{NO}C_{NO_{2}}\theta$
NH_3_ SCR with NO_2_	4 NH_3_-S + 3 NO_2_ → 3.5 N_2_ + 6 H_2_O + 4 S	$R_{7}=A_{7}exp\left(\frac{E_{a,7}}{RT}\right)C_{NO_{2}}\theta$
N_2_O formation by SCR	2 NH_3_-S + 2 NO_2_ → N_2_O + N_2_ + 3 H_2_O + 2 S	$R_{8}=A_{8}exp\left(\frac{E_{a,8}}{RT}\right)C_{NO_{2}}\theta$
